# Fetal growth restriction leads to prolonged nephrogenesis and gene dysregulation in the adult kidney

**DOI:** 10.26508/lsa.202603713

**Published:** 2026-09-23

**Authors:** Sage Timberline, Rachel K Dailey, Wyatt Johnson, Ayyappa Kumar Sista Kameshwar, Jaya Isaac, Kimberly deRonde, Mark Conaway, Aleksandra Cwiek, Kevin M Bennett, Edwin J Baldelomar, Teng Li, Teresa Wu, Matthew R Hoch, Meredith P Schuh, Shalini Indugula, Yoshinori Seki, Masako Suzuki, Kimberly J Reidy, Jennifer R Charlton

**Affiliations:** 1 Division of Critical Care, Department of Pediatrics, University of Virginia School of Medicine , Charlottesville, VA, USA; 2 University of Virginia , Charlottesville, VA, USA; 3 Department of Nutrition, Texas A&M University , College Station, TX, USA; 4 Division of Nephrology, Department of Pediatrics, Children’s Hospital at Montefiore, New York, NY, USA; 5 Division of Nephrology, Department of Pediatrics, University of Virginia School of Medicine , Charlottesville, VA, USA; 6 University of Virginia Health System, Charlottesville, VA, USA; 7 Division of Translational Research and Applied Statistics, Department of Public Health Sciences, University of Virginia School of Medicine, University of Virginia , Charlottesville, VA, USA; 8 Department of Cell Biology, University of Virginia School of Medicine , Charlottesville, VA, USA; 9 Mallinckrodt Institute of Radiology, Washington University School of Medicine, St. Louis, MO, USA; 10 School of Computing and Augmented Intelligence, Arizona State University, Tempe, AZ, USA; 11 Department of Biomedical Engineering, University of Virginia , Charlottesville, VA, USA; 12 Division of Nephrology & Hypertension, Cincinnati Children’s Hospital Medical Center, Department of Pediatrics, University of Cincinnati College of Medicine, Cincinnati, OH, USA

## Abstract

Low birthweight increases chronic kidney disease (CKD) risk. Using a gestational low protein (LP) mouse model, we hypothesized that protein restriction would alter nephrogenesis and adult gene expression. Pregnant CD-1 dams received normal protein (NP) or LP diets. Pups were studied on postnatal days 2–7 to assess the nephron progenitor cell population. Adult mice underwent transdermal GFR measurement and histologic evaluation, bulk RNA sequencing, and cationic-ferritin-enhanced MRI. Nephrogenesis was protracted in LP mice compared with NP mice. Adult LP mice had fewer glomeruli. LP males had lower GFR and more atubular glomeruli without glomerular hypertrophy. LP males demonstrated down-regulated *Gfrα1* and *Aldh1a1*. LP females demonstrated *Gata3* down-regulation. LP offspring exhibited prolonged postnatal nephrogenesis with a lower glomerular number as adults, indicating an extended developmental window did not compensate for their reduced nephron endowment at birth. Suppression of the retinoic acid–GDNF signaling pathway was present in LP males. These findings identify a potential early life therapeutic window to mitigate CKD risk in growth-restricted offspring.

## Abstract (precis)

A mouse model of fetal growth restriction demonstrates prolonged nephrogenesis and gene dysregulation in adulthood. These findings link impaired kidney development to disease risk, highlighting late gestational and early postnatal periods as therapeutic windows during active nephrogenesis to prevent CKD.

## Introduction

Low birthweight occurs in nearly 15% of births worldwide (1). In humans, a birthweight less than 2.5 kg reflects an adverse intrauterine environment. Low birthweight is also associated with a low nephron endowment, but the mechanisms linking the intrauterine environment to nephron development are poorly understood. Nephron endowment is established during a tightly regulated developmental window, in which there is precise coordination between nephron progenitor maintenance and differentiation with reciprocal signaling from the ureteric epithelium (2). Disruption of this process can lead to permanent low nephron number, a recognized risk factor for hypertension, proteinuria, and chronic kidney disease (CKD) (3, 4).

Short et al recently published a comprehensive framework for understanding how an adverse intrauterine environment affects early kidney development (5 *Preprint*). They demonstrated that gestational protein restriction disrupts fetal nephrogenesis through molecular and cellular alterations in nephron progenitor, ureteric, and stromal cells. They identified dysregulation of pathways in this model critical for nephron formation, including Wnt/B-catenin, Gdnf/Ret, and Notch, accompanied by altered ureteric branching, and reduced progenitor cell proliferation and commitment. Their findings establish that protein restriction during gestation compromises nephrogenesis early, limiting the potential for nephron formation.

Interestingly, several human and animal studies suggest that an adverse intrauterine environment frequently results in premature termination of nephrogenesis. Carpenter et al. demonstrated that, while nephrogenesis can persist postnatally in preterm human infants, it ceases earlier than term-equivalent postmenstrual age, indicating that nephron progenitor depletion is accelerated in the extrauterine environment (6). Cerqueira et al showed that in utero exposure to maternal diabetes in mice impairs early nephron progenitor differentiation, with failure to develop into mature nephrons (7). We demonstrated that preterm birth induces premature differentiation resulting in nephron progenitor depletion and early cessation of nephrogenesis (8). Together, these studies suggest that developmental stress can alter nephrogenesis and disrupts the balance between progenitor maintenance and differentiation. While Short et al. evaluated changes in nephrogenesis in the growth-restricted model, their latest time point examined was 2 d after birth, which was prior to the end of nephrogenesis (5 *Preprint*). Here, we examine later developmental time points in the same model of gestational restriction to determine the timing of nephrogenesis cessation. Determining the duration of nephrogenesis in this setting has important translational value because this window not only represents the period of greatest risk for additional environmental harm but also represents an opportunity for postnatal (PN) interventions to increase nephron endowment.

**Figure 1.**
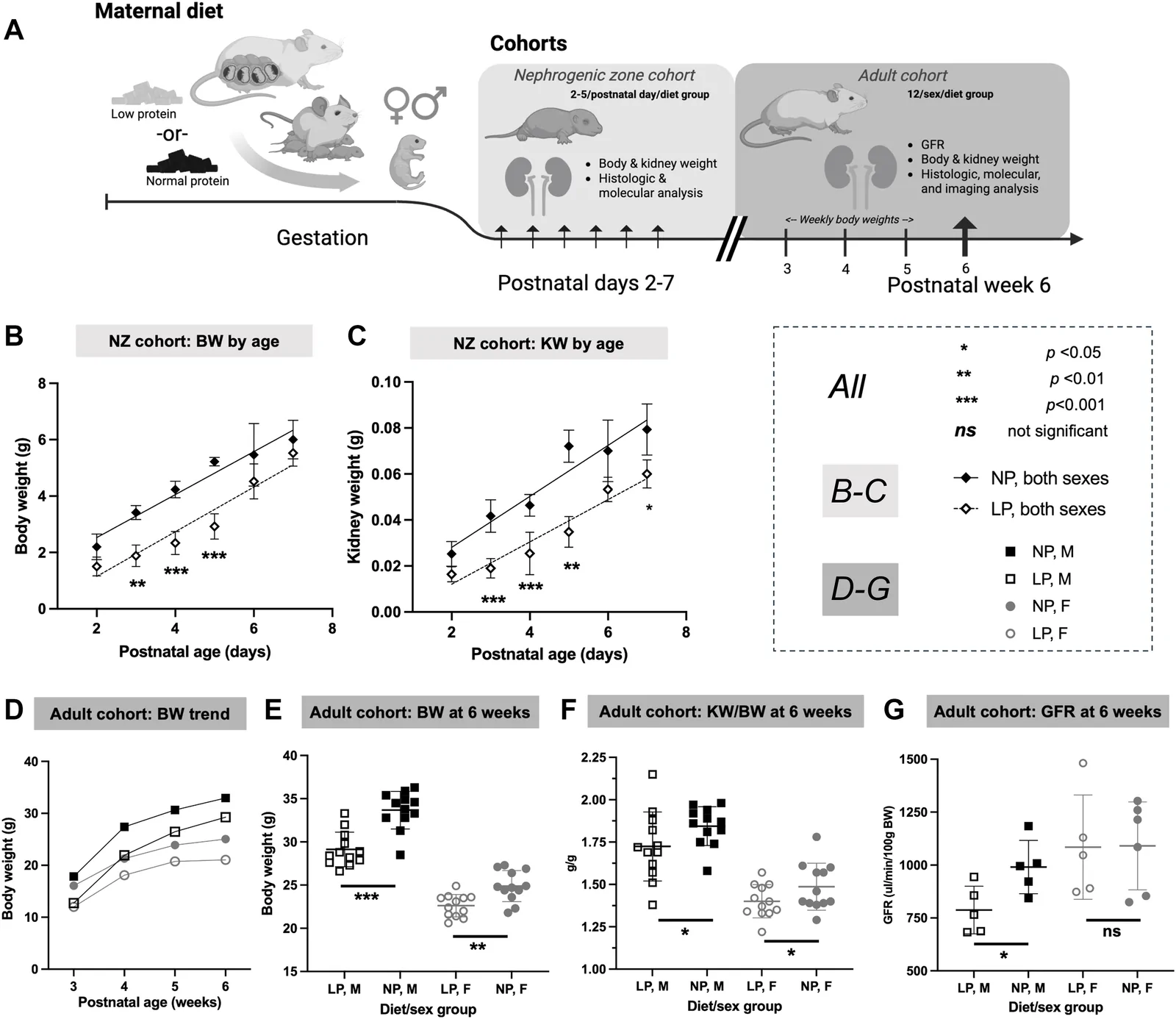
Experimental design and model validation. **(A)** Maternal diet containing low or NP, with offspring, including males and females, divided into cohorts, representing the two general timepoints of interest. **(B, C)** Average body weight (BW) and kidney weight (KW) of pups in the nephrogenic zone (NZ) cohort by postnatal day (PN). Data are presented as mean ± SD. LP pups had lower BW at PN 3, 4, and 5, and lower KW at PN 3, 4, 5, and 7. *n* = 4–6 per diet group per day*. **(D)** Average BW of NP and LP groups in the adult cohort by postnatal week. For each sex and at each time point, BW was lower in LP groups. *n* = 12 per diet group per sex*. **(E, F, G)** BW, KW/BW ratio, and GFR of animals in the adult cohort at 6 wk. Data are presented as mean ± SD. LP groups of both sexes had lower BW and lower KW/BW ratio compared with NP groups of both sexes. LP males also had lower GFR than NP males, while LP and NP females were not different. *n* (BW and KW/BW) = 12 per diet group per sex, *n* (GFR) = 5–6 per diet group per sex*. *LP, low protein; NP, normal protein; M, male; F, female; KW, kidney weight. BW, body weight; NZ, nephrogenic zone; GFR, glomerular filtration rate.* *See Table S7 for all counts.

Low nephron endowment is a characteristic feature after gestational protein restriction, but the potential for permanent molecular changes in the adult kidney has not been established. To understand how impaired nephrogenesis is linked to later susceptibility to acute kidney injury and CKD, it is important to understand whether alterations in early development leave persistent transcriptional signatures that functionally change the kidney after development ends. Data from living kidney donors show that substantial nephron loss does not inevitably lead to CKD, highlighting that nephron number alone may be insufficient to explain long-term vulnerability (9). Prior animal work has demonstrated reduced repair capacity and increased susceptibility to renal insults in those with low nephron endowment (10, 11). These findings support that developmental programming in this setting alters intrinsic pathways in the kidney, leaving it more vulnerable to second “hits” such as ischemia, nephrotoxins, hyperglycemia, or hypertension, rather than simply reducing nephron number (12). For this reason, we sought to characterize the kidney molecular phenotype and developmental trajectory of mice following gestational protein restriction. We hypothesized that low birthweight (LBW) in these offspring would be driven by a truncated period of nephrogenesis and would result in transcriptomic alterations that predispose the adult kidney to future disease. To test this, we exposed pregnant mice to either a low protein (LP) or normal protein (NP) diet, a well-established model in rodents (13). We evaluated the short-term effects on the duration of nephrogenesis in the offspring and investigated the long-term impact on kidney structure and gene expression at 6 wk of age (Fig 1A). This work aims to determine the duration of nephrogenesis and define the adult molecular phenotype associated with growth restriction, laying the groundwork for understanding the increased risk of CKD in those with low nephron endowment.

## Results

Pups were weighed daily from PN days 2–7. Weights in the pups were not different by sex; therefore all animals were analyzed in aggregate. LP pups had lower body weight at PN days 3, 4, and 5 (PN3: LP 1.9 ± 0.4 g versus NP 3.5 ± 0.4 g, *P* < 0.01; PN4: LP 2.4 ± 0.4 g versus NP 4.2 ± 0.2 g, *P* < 0.001; PN5: LP 3.1 ± 0.2 g versus NP 5.1 ± 0.1 g, *P* < 0.001; Fig 1B), and lower kidney weight at PN days 3, 4, 5, and 7 (PN3: LP 0.019 ± 0.004 g versus NP 0.047 ± 0.007 g, *P* < 0.001; PN4: LP 0.023 ± 0.007 g versus NP 0.050 ± 0.002 g, *P* < 0.001; PN5: LP 0.036 ± 0.008 g versus NP 0.073 ± 0.008 g, *P* < 0.01; PN7: LP 0.060 ± 0.006 g versus NP 0.079 ± 0.011 g, *P* = 0.02; Fig 1C), though their kidney weight normalized to body weight (KW/BW) was only lower at PN day 3.

Offspring in the adult cohort were weighed weekly after weaning (Fig 1D). Weights were different by sex; therefore, data were analyzed within each sex. At every time point and within each sex, the LP group had lower body weight than the NP group. At 6 wk, LP males weighed significantly less than NP males (29.1 ± 2.0 g versus 33.7 ± 2.2 g, *P* < 0.0001), as did LP females compared with NP females (22.6 ± 1.8 g versus 24.9 ± 1.3 g, *P* < 0.01; Fig 1E). LP males also had lower KW/BW than NP males (1.7 ± 0.2 g versus 1.8 ± 0.1 g, *P* = 0.04), as did LP females compared with NP females (1.4 ± 0.1 g versus 1.5 ± 0.1 g, *P* = 0.047; Fig 1F). Together, these results demonstrate that LP pups were smaller than NP pups after birth, and remained smaller than NP animals in adulthood, without catch-up growth after switching to the NP diet at the time of weaning. LP males had a significantly lower GFR compared with NP males (787 ± 112 versus 949 ± 152 µl/min/100 g BW, *P* = 0.04), Fig 1G and Table S1). In contrast, no difference in GFR was observed between LP and NP females (1084 ± 247 versus 1090 ± 208 µl/min/100 g BW, *P* = 0.97), consistent with the literature (14).

Table S1. Renal structural characteristics and GFR in adult male and female offspring following gestational protein restriction.

### Prolonged duration of nephrogenesis

**Figure 2.**
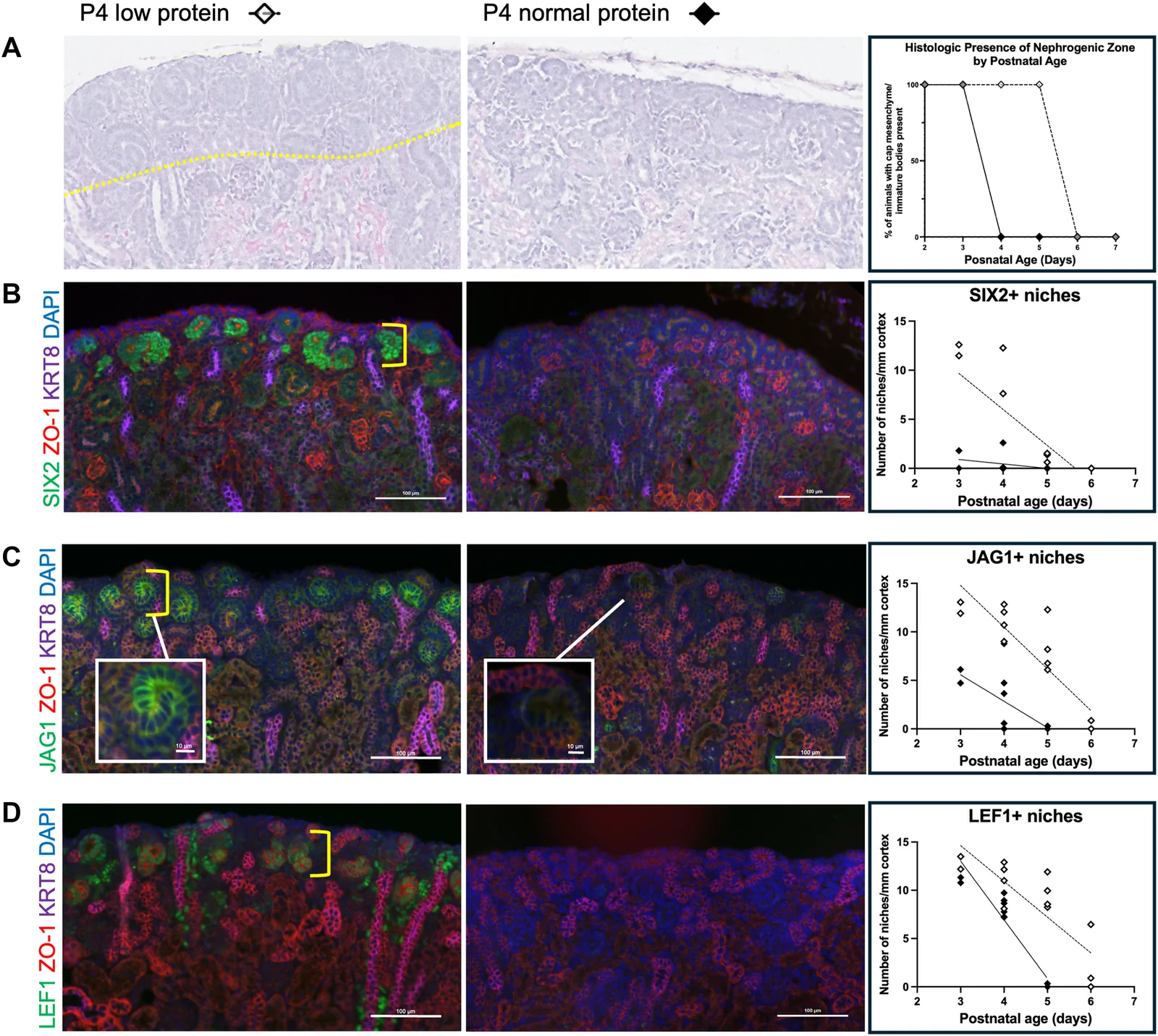
Histologic and molecular evidence of relatively truncated nephrogenesis in LP pups. **(A)** Histologic evaluation of the nephrogenic zone, demonstrating persistence of cap mesenchyme and presence of immature bodies in LP animals at PN 4, compared with absence in NP animals. The border of the nephrogenic zone is indicated with a yellow dotted line. *n =* 4–6 per diet group per day*. **(B, C, D)** Molecular evaluation of the nephrogenic niche using immunofluorescence to assess for presence of SIX2, JAG1, and LEF1. Representative images from PN 4 demonstrate persistent expression of markers in LP pups, and faint or absent expression in NP pups. Individual niches are indicated with yellow brackets. Graphs demonstrate persistent expression of markers in LP pups through PN 6, and low to absent expression of markers in NP pups by PN 4 and 5. *n* = 2–6 per diet group per day*. Scale bars, 100 µm in (B, C, D), and 10 µm in magnified insets. *LP, low protein; NP, normal protein*; *PN, postnatal day*. *See Table S7 for all counts*.*

**Figure S1.**
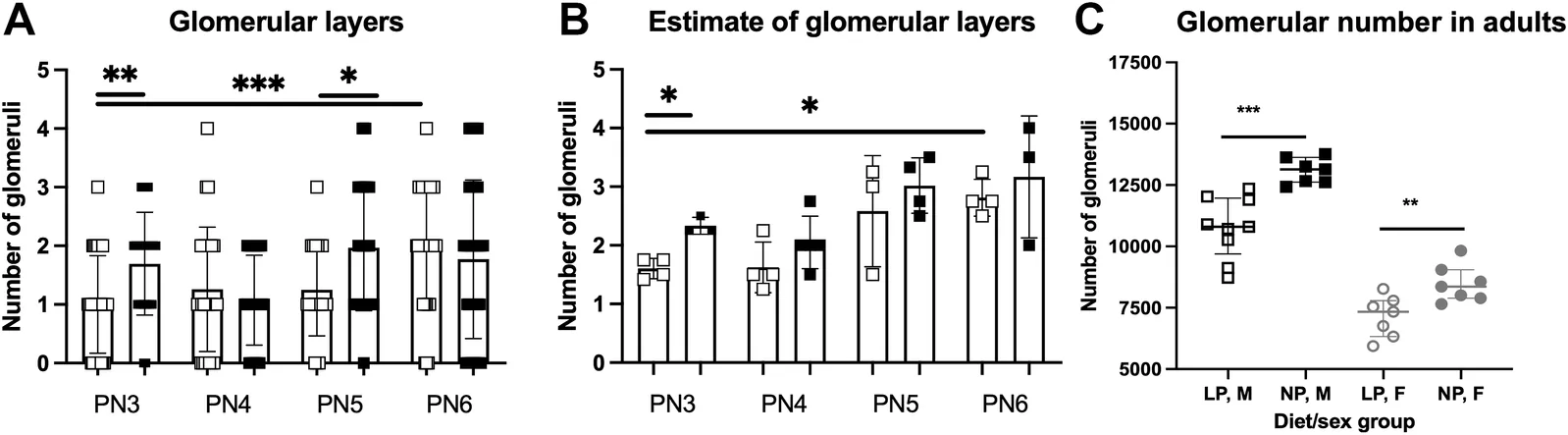
Mature glomerular number from neonates and adults. **(A)** Lines were drawn from medulla to capsule to capture the number of glomerular layers. **(B)** Given the variability of cortex visualized on these small kidneys, a second method was to estimate the number of layers on average throughout the kidney done by manual inspection. With both methods, the trend was the same, the number of mature glomeruli was lower in the low protein (LP) group compared with normal protein (NP) at postnatal (PN) day 3. Over time, there was an increase in mature glomeruli in the LP group. **(C)** At 6 wk, the adult LP group demonstrate a lower glomerular number in both sexes than the NP group. * = *P* < 0.05; ** = *P* < 0.01; *** = *P* < 0.001; *LP, low protein; NP, normal protein; PN, postnatal days*.

Studies have demonstrated a lower nephron number in growth restricted animals. We therefore examined the duration of nephrogenesis and process of nephron differentiation using markers for nephron progenitors (SIX2) and more differentiated structures (JAG1 and LEF1) during this time to better understand when and how these animals reach their full nephron endowment. Comma-shaped and S-shaped immature bodies and the structure of the cap mesenchyme continued to be detectable through PN5 and were not present on PN6 in the LP group (Fig 2A), whereas these were not present by PN4 in the NP group. Based on immunofluorescent staining, there was continued expression of SIX2 on PN4 and 5 in the LP group, compared with low or absent expression of SIX2 in the NP group, shown in a representative image in Fig 2B. There was persistent protein expression of JAG1+ nascent nephrons and strong LEF1 staining through PN6 in the LP pups, whereas expression of JAG1 and LEF1 was no longer present by PN4 in NP pups. Linear regression analysis of expression over time showed significant differences in slopes (Fig 2B and C; SIX2, *P* = 0.01; JAG1, *P* = 0.04) and/or intercepts (Fig 2D; LEF1, *P* < 0.0001). Linear regression also predicted a difference in when the mean number of niches/mm of cortex would fall below 1 for each group. For SIX2, the LP group was predicted to take 5.4 PN days to have fewer than 1 niche/mm cortex, but the NP group was predicted to take 2.8 PN days (*P* = 0.03). For JAG1, the LP group was predicted to take 6.2 PN days, whereas the NP group was predicted to take 4.7 PN days (*P* = 0.056). For LEF1, the LP group was predicted to take 6.7 d, but the NP group was predicted to take 5.0 d (*P* = 0.02). Contrary to our hypothesis that the duration of nephrogenesis would be shorter in the LP animals, all measures of duration of nephrogenesis were longer in the LP group, with normal but delayed differentiation. At PN3, the LP group had fewer mature glomeruli than the NP group (Fig S1A–C). From PN3 to PN6, there was an increase in the number of mature glomeruli in the LP group. These data suggest that the final glomerular number was lower in the LP because nephron endowment just after birth was lower in the LP group compared with NP group and a prolonged duration of nephrogenesis in the LP group could not overcome this deficit.

### Structural alterations in adult kidneys exposed to LP

**Figure 3.**
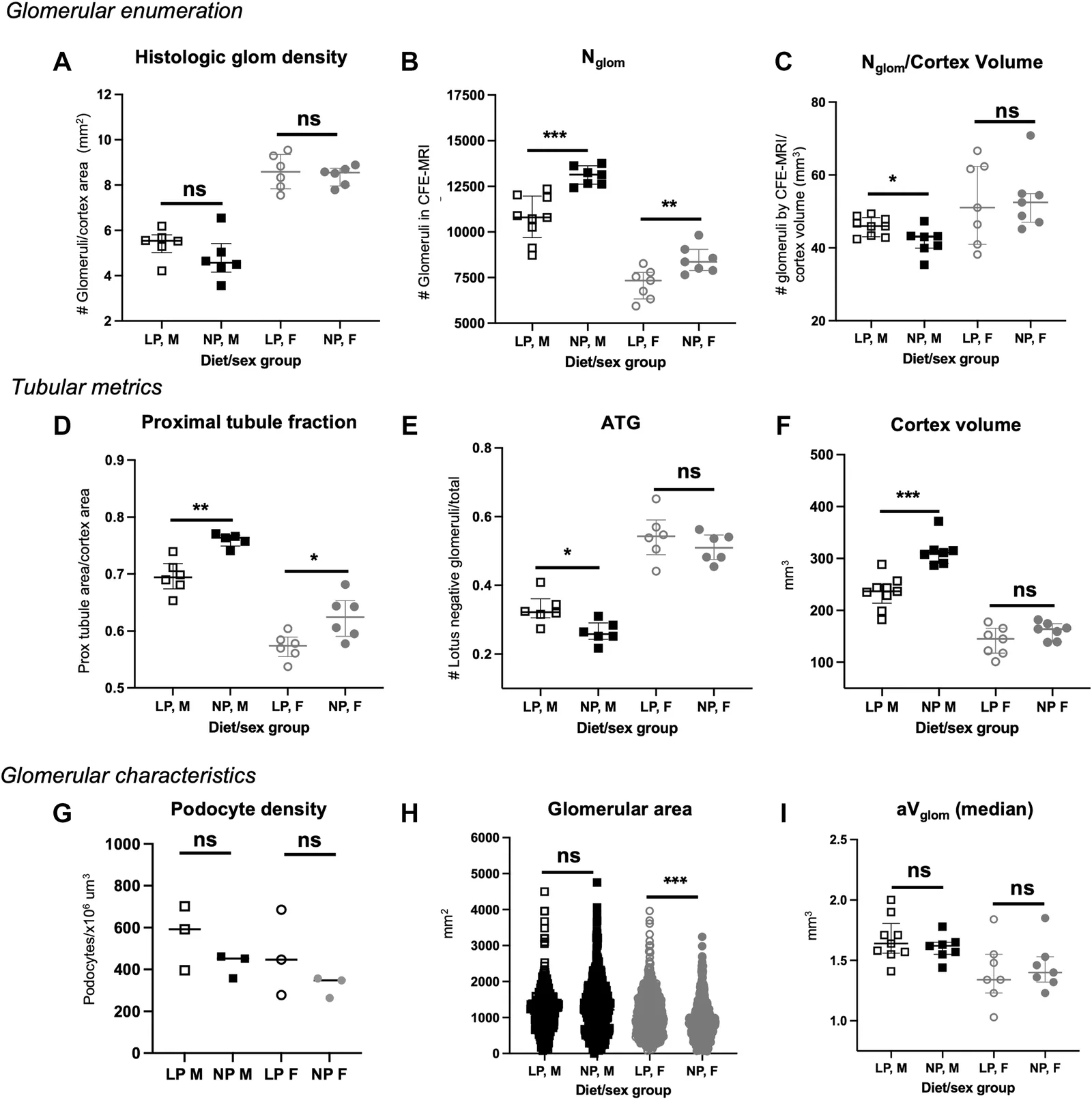
Glomerular enumeration, tubular metrics, and glomerular characteristics of adult LP and NP animals using histology and CFE-MRI. **(A)** Histologic glomerular density (number of glomeruli per 2D section/cortex area) was not different in LP compared with NP groups, though it was lower in males compared with females. *n* = 6 per diet group per sex*. **(B)** N_glom_ (number of glomeruli in each 3D CFE-MRI, measured by UDHoG deep learning method) was lower in LP compared with NP groups, and was also lower in females compared with males. *n* = 7–9 per diet group per sex*. **(C)** N_glom_ per cortex volume was higher in LP males compared with NP males, but was not different in LP females compared with NP females, suggesting cortex loss is greater than N_glom_ loss in males. *n* = 7–9 per diet group per sex*. **(D)** Proximal tubule fraction was lower in LP compared with NP groups, and was also lower in females compared with males. *n* = 5–6 per diet group per sex*. **(E)** Atubular glomeruli (ATG) fraction was higher in LP males compared with NP males but was not different in LP females compared with NP females. ATG fraction was also lower in males compared with females. *n* = 5–6 per diet group per sex*. **(F)** Cortex volume was lower in LP males compared with NP males, but was not different in LP females compared with NP females. Cortex volume was higher in males compared with females. *n* = 7–9 per diet group per sex*. **(G)** Podocyte density was not different by diet or sex groups. *n* = 10 glomeruli per 2D section per animal × 4–6 per diet group per sex*. **(H)** Glomerular area was not different between LP and NP males, but was larger in LP females compared with NP females. *n* = all glomeruli per 2D section per animal × 4–6 per diet group per sex*. **(I)** aV_glom_ (average volume of glomeruli in each 3D CFE-MRI, measured by UDHoG deep learning method) was measured for each animal, and the median of all animals in a group was used to compare across groups. aV_glom_ was not different by diet or sex groups. *n* = 7–9 per diet group per sex*. *LP, low protein; CFE-MRI, cationic-ferritin enhanced-magnetic resonance imaging; NP, normal protein; M, male; F, female.* * = *P* < 0.05; ** = *P* < 0.01; *** = *P* < 0.001; ***ns*** = not significant. *See Tables S1 and S7 for all counts*.*

To determine the effect of maternal protein restriction on glomerular and tubular structural changes in the kidney, both histologic and CFE-MRI methods were used to assess differences in the LP compared with NP groups. Detailed data summary has been provided in Table S1. Glomerular density by histologic assessment was not different in LP animals compared with NP (LP males: 5.5 [IQR 5.0–5.8] versus NP males: 4.6 [IQR 4.2–5.4] glomeruli/mm^2^, *P* = 0.22; LP females: 8.6 [IQR 7.8–9.4] versus NP females: 8.5 [IQR 8.0–8.7] glomeruli/mm^2^, *P* = 0.66; Fig 3A), but N_glom_ by CFE-MRI was higher in NP animals (Fig 3B). LP males had 18% fewer glomeruli than NP males (10,799 [IQR 9,695–11,966] versus 13,142 [IQR 12,616–13,624], *P* < 0.001), and LP females had 16% fewer glomeruli than NP females (7,339 [IQR 6,329–7,788] versus 8,363 [IQR 7,892–9,046], *P* < 0.01). LP males also had higher N_glom_ per cortex volume than NP males (46 [IQR 43–48] versus 43 [IQR 40–43] glomeruli/mm^3^, *P* < 0.05), suggesting that cortex volume is not only smaller, but disproportionately smaller relative to glomerular number in LP males (Fig 3C). LP animals had lower PT fraction compared with NP animals in both sexes (LP males: 0.69 [IQR 0.67–0.71] versus NP males: 0.76 [IQR 0.74–0.76], *P* < 0.01; LP females: 0.57 [IQR 0.55–0.58] versus NP females: 0.65 [IQR 0.59–0.65], *P* < 0.05; Fig 3D). There were more ATG in LP males compared with NP males (0.32 [IQR 0.31–0.36] versus 0.26 [IQR 0.24–0.29], *P* < 0.05), but no difference was observed in females (LP: 0.54 [IQR 0.49–0.59] versus NP: 0.51 [IQR 0.48–0.55], *P* = 0.35; Fig 3E). Cortical volume was lower in the male LP group (Fig 3F). Podocyte density and glomerular area were not different between male LP and NP groups (Fig 3G and H). Importantly, aV_glom_ was not different between LP and NP groups (Fig 3I). In contrast, glomerular area was higher in the LP females when measured on a 2D section, suggesting the importance of evaluating the whole kidney for glomerular volume (Fig 3H). Overall, LP animals had fewer glomeruli, similar average volume and podocyte density, with lower PT fraction and cortex volume, suggesting fewer tubules.

Histologic analysis and CFE-MRI were used to identify sex differences in structural changes in glomeruli and tubules in LP and NP groups. By histology, males in each group had a larger kidney cortex area compared with females, but no difference in the absolute glomerular count, and therefore a lower glomerular density. Males also had a higher PT fraction and a smaller proportion of ATG, as well as a larger glomerular area compared with females. Similarly, by CFE-MRI, males had larger kidney and cortex volume and higher N_glom_. Only LP males had larger aV_glom_ than LP females, while NP males and females were similar. N_glom_ and aV_glom_ were somewhat correlated in NP females (R^2^ = 0.73), less so in LP females (R^2^ = 0.54, respectively), and not at all in NP and LP males (R^2^ < 0.1). There was no significant difference in podocyte density between males and females.

### Transcriptomic alterations in adult kidneys exposed to LP

Bulk RNA-seq analysis was performed using both an unbiased (traditional) and a candidate-gene approach. Initial sex classification was performed based on phenotypic observation and subsequently validated using Y-chromosome-specific gene expression analysis (*Uty* and *Ddx3y*). A sample, initially identified as female, was reclassified as male based on Y-chromosome-specific gene expression and was excluded from all downstream analyses to maintain cohort consistency, leaving five high-quality samples in LP, male; NP, male; and LP, female group and three samples in NP, female group. Since we observed a clear branch separation in hierarchical clustering analysis, we performed RNA-seq analysis stratified by sex. Candidate genes were chosen *a priori* and organized into the categories of (1) kidney development, (2) nephron structure, and (3) pathways, as previously published (8).

**Figure 4.**
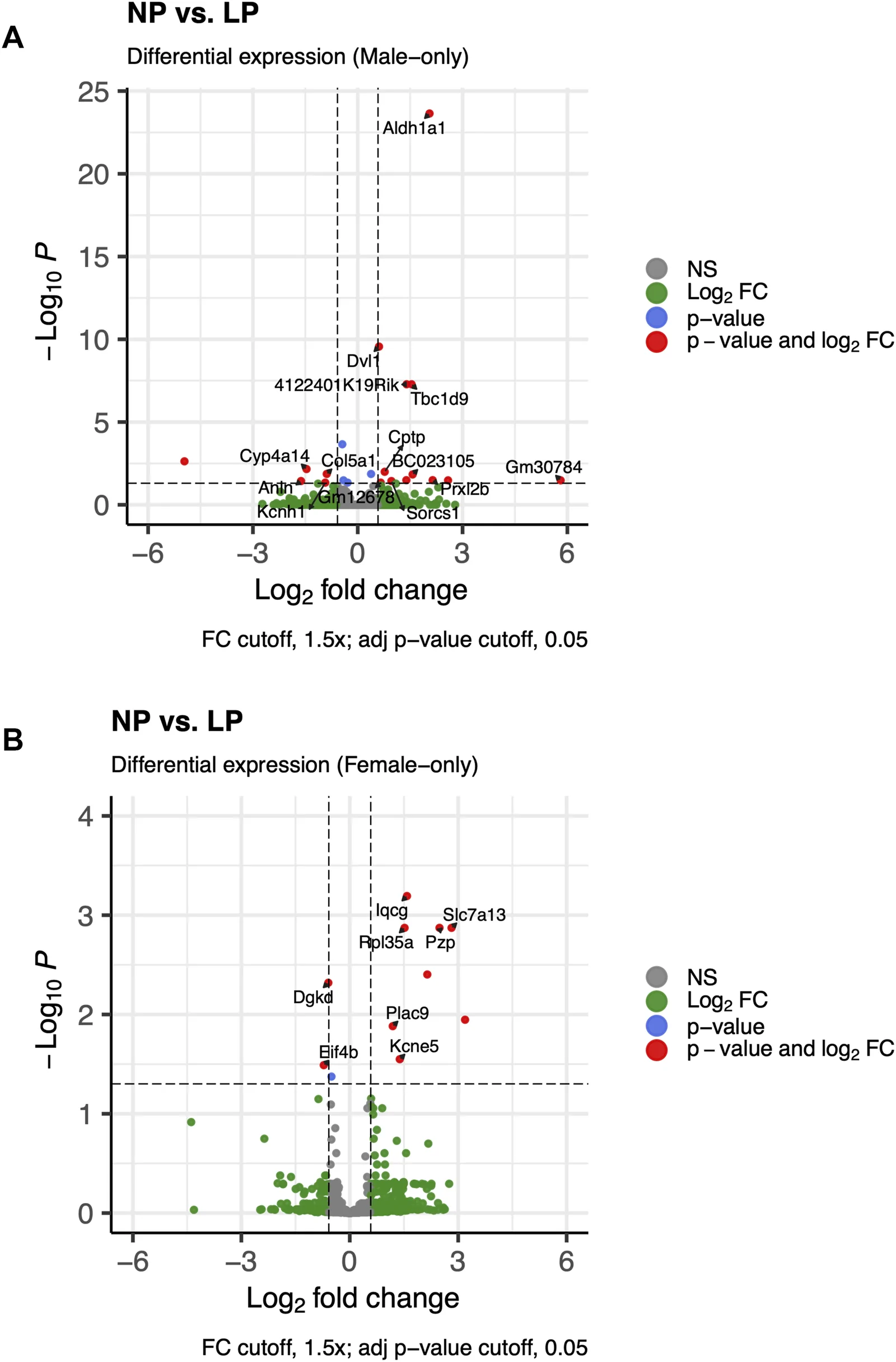
Sex-specific differential gene expression (DEG) in kidneys from offspring exposed to a maternal low-protein diet. Volcano plots showing differential gene expression from bulk RNA-seq analysis comparing kidneys from offspring exposed to a normal protein (NP) versus low protein (LP) maternal diet. Analyses were performed separately for (A) males (top) and (B) females (bottom). The x-axis represents log_2_ fold change (NP versus LP) and the y-axis shows −log10 adjusted *P* value. Dashed vertical lines indicate the fold-change cutoff (1.5×), and the dashed horizontal line indicates the adjusted *P* value threshold (0.05). Genes meeting both statistical significance and fold-change criteria are shown in red, genes significant by *P* value only in blue, genes passing fold-change threshold only in green, and nonsignificant genes in gray. Selected differentially expressed genes are labeled. Note that the y axis scales differ between the male and female volcano plots to reflect the markedly different ranges of log2 fold change observed in each dataset. These results demonstrate a stronger transcriptional response to maternal protein restriction in male kidneys compared with females.

**Figure S2.**
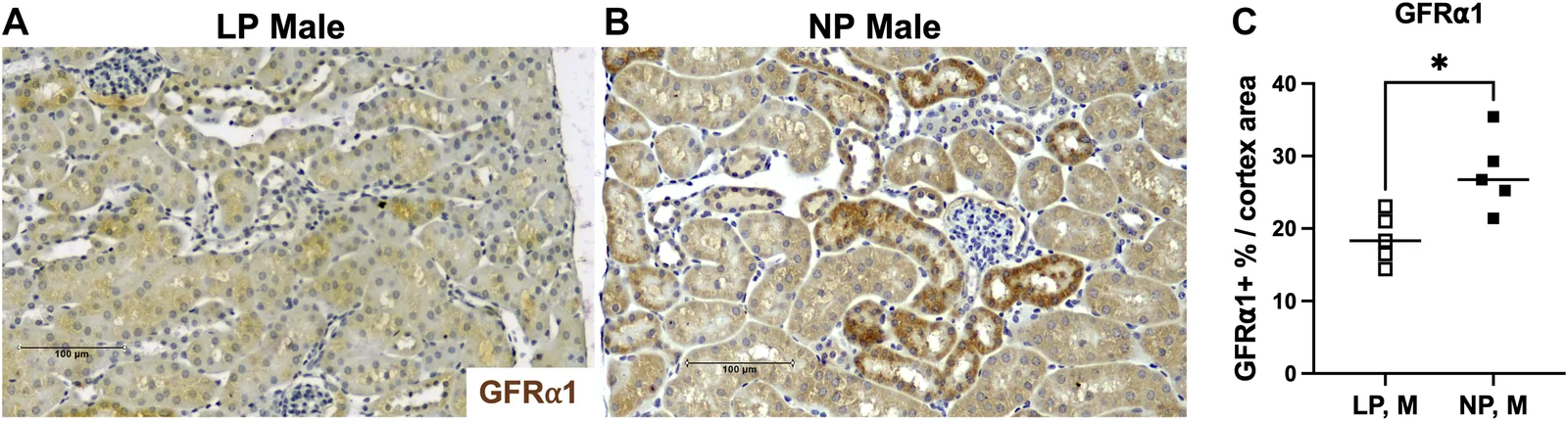
Reduced cortical GFRα1 expression in kidneys from low-protein male offspring. Representative immunohistochemical staining for GFRα1 in kidney cortex sections from male offspring exposed to a low protein (LP, A) or normal protein (NP, B) maternal diet. NP males exhibit stronger and more extensive GFRα1-positive staining within cortical tubular structures compared with LP males, where staining appears reduced. **(C)** Quantification of GFRα1-positive area expressed as a percentage of total cortical area shows significantly lower GFRα1 signal in LP males compared with NP males. Each point represents an individual animal, and horizontal bars indicate mean values. *P < 0.05. scale bar = 100 μm.

From the unbiased approach with sex stratification, several differentially expressed genes (DEGs) were identified. We identified four up-regulated DEGs and 11 down-regulated DEGs in LP males compared with NP (Table S2 and Fig 4A). Down-regulated genes of note included *Gfr⍺1*, *Aldh1a1*, and *Dvl1*. We confirmed the lower expression of GFR⍺1 in LP males by immunohistochemistry (Fig S2A–C). In females, two DEGs were up-regulated in the LP group, and seven, including *Iqcg*, *Pzp*, and *Slc7a13*, were down-regulated in LP compared with NP groups (Table S3 and Fig 4B). The broader y-axis range in the male volcano plot reflects substantially larger log_2_ fold changes than those observed in females, indicating that although both sexes exhibited differentially expressed genes, the magnitude of transcriptional changes was greater in males.

Table S2. Differentially expressed genes in LP compared to NP males.

Table S3. Differentially expressed genes in LP compared to NP females.

For the candidate-gene approach, samples were again stratified by sex. In the kidney development category, genes including *Gfr⍺1, Ffg10,* and *Pax8* were down-regulated in male LP animals, and *Etv5* and *Osr2* were up-regulated in female LP animals, compared with male and female NP groups (Table 1). In the nephron structure category, *Foxd1*, and *Trpv5* were up-regulated in male LP animals, and *Gata3, Mgp, and Trpv6* were down-regulated in female LP animals, compared with male and female NP groups. In the pathways category, females had no DEGs that were significant after FDR correction. In males, *Hif1an, Egln3, and Col5a1* were up-regulated in LP compared with NP animals, and *Dvl1* and *Aldh1a1* were down-regulated. Detailed expression of all significant genes at *P* < 0.05 are listed in Table S4 and all the genes analyzed are listed in Table S5.

**Table 1.** Differentially expressed genes after multiple comparison adjustment.

Sub category	Gene	Difference: LP - NP	SE	Lower 95% CI	Upper 95% CI	*P* value
Kidney development						
Males						
Ureteric bud	*Gfr⍺1*	−2.418	0.226	−2.940	−1.897	<0.001
Metanephric mesenchyme	*Fgf10*	−1.592	0.377	−2.463	−0.722	0.003
Renal vesicles	*Pax8*	−0.231	0.065	−0.381	−0.080	0.008
Females	​	​	​	​	​	​
S-shape	*Etv5*	0.469	0.020	0.416	0.521	<0.001
	*Osr2*	0.508	0.114	0.216	0.800	0.007
Nephron structure						
Males						
Cortical/medullary stroma	*Foxd1*	0.437	0.106	0.192	0.682	0.003
Distal tubule	*Trpv5*	0.507	0.132	0.204	0.811	0.005
Females	​	​	​	​	​	​
Mesangial	*Gata3*	−0.468	0.088	−0.694	−0.241	0.003
Cortical/medullary stroma	*Mgp*	−0.446	0.076	−0.641	−0.252	0.002
Distal tubule	*Trpv6*	−0.688	0.166	−1.114	−0.261	0.009
Pathways	​	​	​	​	​	​
Males						
Oxygen sensing/hypoxia	*Hif1an*	0.09	0.018	0.048	0.132	<0.001
	*Egln3*	0.598	0.176	0.193	1.003	0.009
Wnt signaling	*Dvl1*	−0.421	0.043	−0.52	−0.322	<0.001
Vitamin A pathway	*Aldh1a1*	−1.439	0.131	−1.742	−1.136	0.001
Fibrosis	*Col5a1*	0.593	0.134	0.285	0.902	0.002

Table S4. Candidate differentially expressed genes in males and females with significance at *P* < 0.05.

Table S5. All candidate genes analyzed in males and females with statistical results.

**Figure S3.**
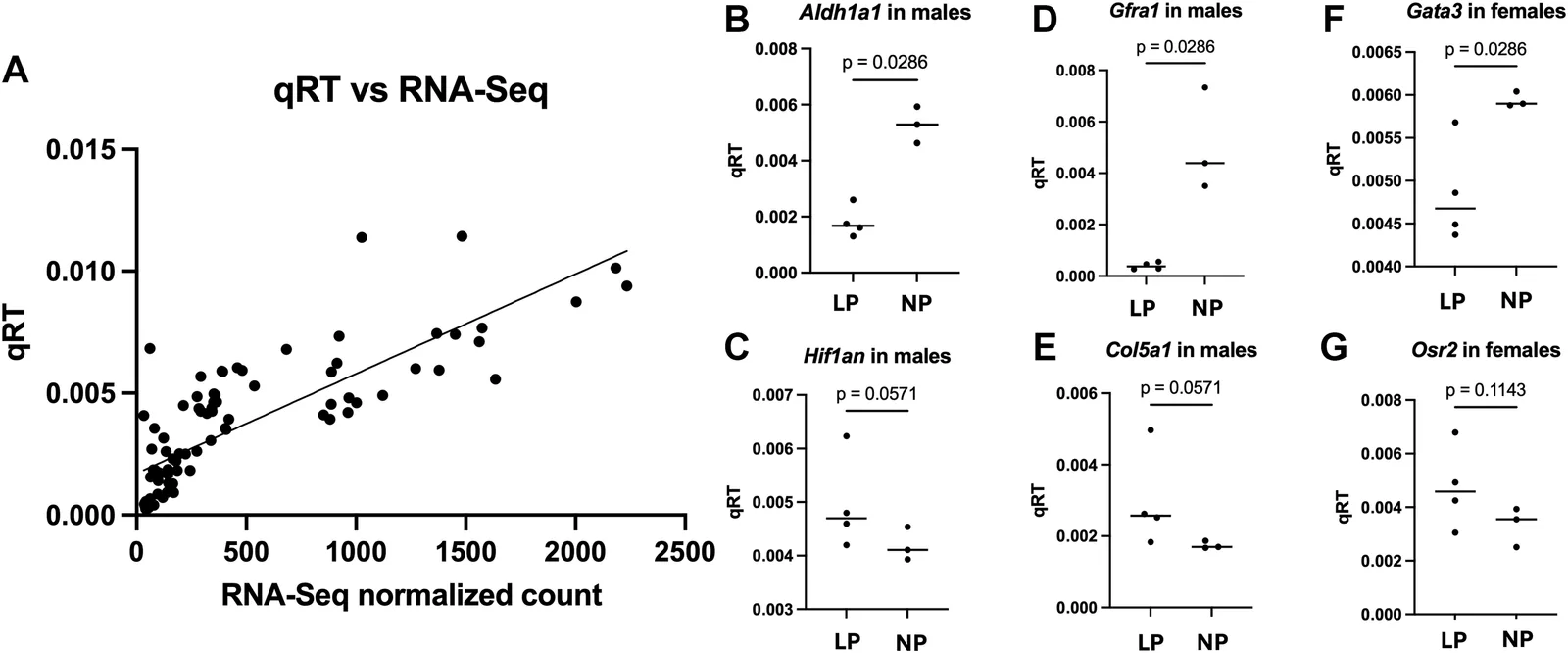
Validation of bulk RNA-Sequencing. qRT was performed to validate the RNA-Sequencing analysis. qRT was strongly correlated with RNA-Seq normalized counts with R^2^ = 0.66, *P* < 0.0001 (A). In the males, *Aldh1a1* (B), *Hif1an* (C), *Gfr*⍺*1* (D), and *Col5a1* (E) were significantly different or trended toward significantly different between the LP and NP groups. In the females, *Gata3* (F) was lower in the LP group than NP group with *Osr2* trending toward significance (G).

Validation by qRT-PCR was performed on 14 samples and included nine genes from the female group and six in the male group. The qRT values strongly correlated with bulk RNA-sequencing normalized counts (*P* < 0.0001, Fig S3A). Validated genes from both males and females are included in Fig S3B–G.

## Discussion

This study expands on prior studies of growth restriction models (15, 16), demonstrating a slower, prolonged nephrogenesis, and an adult kidney with a permanently altered molecular phenotype. During active nephrogenesis, LP kidneys exhibited persistence of histologic and molecular evidence of nephrogenesis, as well as a slower increase in number of mature glomeruli, indicating a delayed but less effective process. This delay was accompanied by a reduced nephron endowment, demonstrated in both the early PN and adult periods, and by structural and functional sequelae including reduced PT fraction in both sexes, and a lower GFR and increased fraction of ATG in males. Bulk RNA-sequencing of adult kidneys revealed a sex-specific molecular signature in several genes important in kidney development, particularly ureteric bud branching morphogenesis. LP males showed suppression of *Gfr⍺1* and *Aldh1a1*, implicating lasting alteration of GDNF/RET and retinoic acid signaling, while LP females showed decreased expression of *Gata3*. As nephrogenesis is complete in the early PN period, these adult transcriptional differences are unlikely to represent ongoing developmental signaling, but either constitute a persistent imprint of pathway reprogramming or signal that these genes may have other roles in the adult kidney. To our knowledge, this is the first study to evaluate both the duration of nephrogenesis and the adult molecular phenotype in the same growth restriction model, linking prolonged but slowed nephrogenesis with permanent structural, functional, and transcriptional alterations in the mature kidney.

The term “adverse maternal environment” includes many exposures during gestation that can affect the delicate balance between the maintenance of a nephron progenitor niche and differentiation into functional nephrons. Our hypothesis that gestational protein restriction would truncate nephrogenesis was informed by prior work showing that preterm birth can precipitate premature nephron differentiation and disrupt the balance between differentiation and self-renewal (6, 7, 8). Our group demonstrated that preterm birth induced loss of SIX2^+^ nephron progenitor cells. Similarly, Cerqueira et al showed that exposure to maternal diabetes induced impaired differentiation in the offspring with increased SIX2 expression in the setting of decreased JAG1 and LEF1, implicating aberrant canonical Wnt/B-catenin and Notch pathways signaling (7). In contrast, this gestational protein restriction model demonstrated persistence of SIX2, JAG1, and LEF1 expression, with a longer nephrogenic window despite a lower nephron number in mice exposed to LP. Notably, JAG1 and LEF1 are downstream components of Notch and canonical Wnt signaling, respectively, suggesting that these differentiation pathways remain active for a longer duration in LP pups but nephron differentiation may operate at a reduced or inefficient pace. Together, these studies suggest that different prenatal stressors share the downstream phenotype of reduced nephron endowment through divergent mechanistic pathways. While inflammatory stress, like that associated with preterm birth, may accelerate differentiation and lead to early progenitor exhaustion, chronic nutrient restriction may preserve progenitor identity and differentiation signaling over an extended period, but with delayed and ultimately reduced nephron production.

Short et al. eloquently assessed the early structural and molecular changes of offspring exposed to the maternal LP diet used in this study (5 *Preprint*). Using the same mouse strain and degree of protein restriction, they studied offspring at E14.5, PN0, and PN2 and demonstrated impaired branching morphogenesis and nephron progenitor balance. NPC, UB, and cellular proliferation were assessed using wholemount stained kidneys. The LP group exhibited lower proliferation in both the NPC and UB at E14.5 and PN0, but by PN2 there was no difference, suggesting this adverse maternal environment affects the NPC’s ability to commit to formation of a nephron. Our study demonstrates that the window for intervention may be longer than initially appreciated. The molecular markers of nephrogenesis, particularly the presence of SIX2, persisted until PN5, suggesting a longer PN window for therapeutic intervention or susceptibility to injury.

There are limited data regarding potential therapeutic strategies that have translational potential for maximizing nephron endowment in growth restriction models. Cullen-McEwen et al. showed that shifting maternal nutrition to a high-fat (LP-HF) diet during early lactation can rescue the nephron deficit induced by gestational protein restriction (17). At PN3, LP-HF animals showed more SIX2^+^ self-renewing structures than normal diet offspring, and the authors suggest that HF diet may prolong nephrogenesis. However, the difference in SIX2^+^ self-renewing structures was non-significantly higher in LP-HF animals compared with LP animals at PN3. In context of our work, it is likely that investigating SIX2^+^ self-renewing structures at PN4-5 would provide more conclusive evidence of the impact of LP-HF diet on nephrogenesis duration. Pezzotta et al. demonstrated that SIRT3, which has an important role in dictating nephron endowment, had impaired renal expression in LP mice (18). Gestational supplementation of nicotinamide riboside, a precursor of SIRT3 co-substrate nicotinamide adenine dinucleotide (NAD+), restored SIRT3 expression and nephron number in LP animals. In another model of growth restriction using reduced uterine perfusion, glomerular number was partially mitigated by maternal tadalafil treatment, a phosphodiesterase inhibitor which has demonstrated promise in improving blood flow in the placenta (19). Together, these studies suggest that mechanism-based approaches could translate to improving nephron endowment if used during an appropriate window of nephrogenesis.

To clarify the connection between low nephron endowment and predisposition to future kidney disease, we analyzed the molecular phenotype of adult kidneys using bulk RNA sequencing. Several studies have demonstrated compromised responses to renal stress and impaired tissue repair in animals with low nephron endowment (20, 21). Zimanyi et al. found that LP male offspring developed significant up-regulation of *Tgfb1* and procollagen III following secondary renal injury from advanced glycation end-products, which was not observed in NP animals (22). Similarly, Kallash et al. found LP rats exposed to unilateral nephrectomy or high salt diet developed albuminuria and evidence of functional and structural damage compared with NP rats (11). In our study, adult kidneys of LP offspring retained differential expression of genes governing nephron development, suggesting a transcriptional imprint on certain pathways that persists well beyond the completion of nephrogenesis. Developmental pathways contribute to adult kidney epithelial differentiation, extracellular matrix remodeling, repair, fibrosis, and maladaptive injury responses. As discussed by Little and by Edeling et al., reactivation of developmental programs in adult kidneys regulate repair and fibrosis, while studies of low-protein diet demonstrate developmental programming leading to low nephron endowment and later kidney risk (20, 21). Two gene regulators of renal developmental pathways were dysregulated in LP males, *Gfr⍺1* and *Aldh1a1. Gfr⍺1* down-regulation was confirmed at both the transcript and protein levels. GDNF/RET signaling, mediated by the obligate co-receptor GFR⍺1, is required for ureteric bud outgrowth and branching, with even partial reductions in Ret pathway activity leading to renal hypoplasia and reduced nephron number (23, 24, 25). Down-regulation of *Gfr⍺1* may lead to impaired nephron induction or maintenance of the progenitor pool (26), and its persistent down-regulation in adults may mark a lasting alteration in this axis. Comparing our data to the Short study, in which most Ret pathway genes were unaffected by LP but both *Gdnf* and the co-receptor *Gfr⍺1* were down-regulated at E14.5, indicates that *Gfr⍺1* suppression seen prenatally in that model persists into adulthood in the males (5 *Preprint*). *Aldh1a1*, a key enzyme in retinoic acid synthesis, is essential for progenitor differentiation and nephron segmentation and helps maintain Ret pathway activity (27, 28). Its reduced expression suggests possible defects in retinoid signaling, which may contribute to both development and repair after injury (27, 28, 29). The convergence of these findings on the intersecting GDNF/RET-retinoic acid axis is notable given that this same axis regulates the progenitor-to-nephron transition whose delay we observed neonatally.

Maternal exposure to LP during gestation has been frequently used to model the effect of developmental programming of the kidney. LP exposure during gestation results in both a low nephron number and long-term risk of hypertension and CKD. Here, glomerular number was reduced in both males (18%) and females (16%), consistent with reports in the literature (30). Interestingly, the LP animals did not have a larger glomerular volume despite a significant reduction in glomerular number. There are several potential reasons for the lack of glomerular hypertrophy, including that the reduction in glomerular number is not large enough to precipitate hypertrophy, 6 wk of age is too early to observe hypertrophy, or there is an inability to undergo hypertrophy in this model. Interestingly, the lower GFR in the LP male cohort suggests that the reduction in glomerular number was significant, so it is more likely that this model may not possess the reserve to undergo significant glomerular hypertrophy.

In addition to glomerular endowment, our histologic analysis revealed signs of tubular compromise. The proximal tubule highly energy dependent and is the principal site of injury in most progressive kidney disease (31). The reduced proximal tubule fraction in all LP offspring therefore could suggest a potential effect on the reabsorptive and metabolic reserve per kidney, independent of nephron number. This structural finding is reinforced at the transcriptional level, where LP males showed altered expression of *Pax8*, a transcription factor required for tubular epithelial differentiation and maintenance, and *Trpv5*, the apical channel mediating active calcium reabsorption in the distal nephron (32, 33, 34). Together, these data suggest that gestational protein restriction may perturb the tubular compartment in addition to the glomerular effect. Collectively, these histologic findings point to a structural picture of impaired tubular mass, disconnected nephrons, and sex-specific vulnerability that parallels the molecular changes.

Sex-specific differences in the molecular and structural phenotypes underscore the complexity of the response to early life protein restriction. Male LP offspring exhibited reduced kidney function and greater signs of maladaptive tubular changes, while females appeared relatively protected. This difference between sexes is consistent with other rodent studies showing that male offspring are often more susceptible to developmental programming of renal and cardiovascular disease.

There are several limitations of this study. First, maternal and offspring food intake was not measured, and differences in caloric intake between groups could have contributed to the observed phenotypes. However, litter size, duration of gestation, and neonatal mortality were not different between groups, suggesting that the maternal nutritional insult was not severe enough to affect gross pregnancy outcomes. The low-protein formulation has a higher sucrose content than the control diet to balance total calories. While we cannot entirely decouple the potential additive stress of elevated sucrose load on embryonic renal microvasculature, the phenotypes observed in this study, including reduced nephron endowment and lower body and kidney weights, closely mirror historic LP literature where complex starches were used as fillers using similar ad libitum feeding designs (15, 35). Future studies using a third, carbohydrate-controlled group (e.g., substituting sucrose with cornstarch or maltodextrin) could be valuable to completely isolate the subtle metabolic cross-talk of sugar and protein in utero. Finally, the evaluation of nephrogenesis duration included a reduction in litter size over PN days 2–7. Thus, it is possible that nutrition per pup increased over that period. However, because pups were collected on the same schedule from both LP and NP litters, any influence of progressively reduced litter size applied equally to both experimental groups at each timepoint. Our central comparison was the difference in nephrogenic markers, so a litter-size effect should not impact the difference we observed. Blood pressure was not directly measured, which precludes a comprehensive assessment of its role in a model that notably lacks compensatory glomerular hypertrophy. Our RNA evaluation used bulk sequencing but lacks single cell resolution to identify the specific cell-types impacted by these potential pathways. However, this bulk sequencing provides the starting point for these molecular evaluations. The dysregulated genes and pathways that have been identified in this study need further evaluation to determine their mechanistic role between the adverse maternal environment of LP exposure and CKD.

In conclusion, this study offers new insights into how maternal protein restriction affects kidney development and long-term health in offspring. Offspring exposed to LP during gestation have a prolonged but delayed nephrogenesis and several genes that are dysregulated into adulthood. These findings refine our understanding of developmental programming and suggest that strategies to support progenitor cell health and signaling during the extended window of nephrogenesis could potentially improve kidney outcomes after growth restriction. The molecular profile of LP males with impairment of the retinoic acid-GDNF axis may explain the ineffective nephrogenesis and the structural and functional changes observed in adult mice. Together, this work provides a foundation for determining whether these molecular signatures increase susceptibility to a more rapid functional decline during aging or following subsequent insults such as acute kidney injury. Furthermore, these findings may facilitate the development of interventions to increase nephron endowment or lower the long-term risk of CKD.

## Materials and Methods

Reagents used in the project are provided in Table S6. The number of animals and litters used in each assessment are shown in Tables S7 and S8. All experiments were performed at the University of Virginia in accordance with the National Institutes of Health Guide for the Care and Use of Laboratory Animals. All procedures involving animals were approved by University of Virginia’s Institutional Animal Care and Use Committee. ARRIVE 2.0 Essential 10 Checklist was used.

Table S6. Key resources table.

Table S7. Experimental group allocation and sample size.

Table S8. Animal numbers per litter.

### Low birthweight mouse model

Gestational protein restriction is a common method used in rodents to study developmental origins of kidney disease (13, 36). Langley-Evans demonstrated the association of gestational protein restriction with hypertension, decreased lifespan, and lower nephron number in rats (37, 38, 39). Subsequently, similar findings have been replicated in mice (15) using diets contain 9% protein (LP) and 20% protein (NP). In this study, gestational protein restriction was achieved using customized diets (Inotiv/Teklad) containing 18% protein (TD.96180, NP) for control animals, and 8% protein (TD.93033, LP) for experimental animals. Diets were formulated using standard purified ingredients to ensure they were strictly isocaloric (3.8 kcal/g) and matched for lipid and macronutrient content. This approach isolates the effects of reduced amino acid intake while minimizing confounding from overall caloric restriction or maternal energy deprivation. The replacement of protein with simple carbohydrates represents the standard formulation used in developmental programming studies using the normal/LP paradigm (15). The detailed diet composition is shown in Table S9.

Table S9. Detailed diet composition.

CD-1 grandparental mice (six females and three males) were purchased from Charles River Laboratories. These mice were maintained on an NP diet and bred to generate the F0 population. F0 females were assigned to either an LP or NP diet at the time of mating and were bred using a trio-mating strategy (two females and one male) with unrelated CD-1 males. Pregnant females were fed *ad libitum* through gestation and lactation on their designated diet, such that differences in total caloric intake between LP and NP dams were minimized by design. The assigned diets were continued throughout gestation and lactation to ensure dietary exposure during the entire period of nephrogenesis (15), which extends into the PN period in mice (Fig 1A). At the time of weaning, all offspring, regardless of maternal dietary group, were transitioned to the NP diet and maintained on it throughout the study. Further information is provided in Table S8.

#### Nephrogenesis evaluation

Previous work has demonstrated that CD-1 mice complete nephrogenesis on PN day 4 (26). To evaluate the duration of nephrogenesis, the LP and NP pups were euthanized via decapitation on PN days 2 through 7 (n = 3–4 per day). The day of birth was recorded as PN day 0. Kidney weights, body weights, and sex were recorded at the time of collection. An additional litter of pups from a timed pregnancy for each diet group was used. This was necessary because differences in gestation length between LP and NP dams could have confounded the interpretation of the timing of nephrogenesis. For timed pregnancies, mating was confirmed via vaginal plug identification and designated as embryonic day 0, allowing PN age to be calculated relative to a known conception date. The duration of gestation was not different between LP and NP dams, allowing differences in the duration of nephrogenesis to be attributed to dietary intervention rather than differences in gestational timing. There was no difference in neonatal mortality between groups. In addition, there were no differences by sex, therefore, data were reported by diet group and PN age. Offspring from the timed pregnancies added up to two pups per PN day, totaling n = 4–6/diet group/day. Starting litter sizes were comparable between groups (LP: 10.3 ± 2.2, NP: 10.7 ± 2.1, *P* = 0.81), and pups were collected on the same schedule from both LP and NP litters (1–2 pups per litter per day), so the progressive reduction in litter size was equivalent across LP and NP groups at each time point. Details are shown in Table S8.

### Histologic evaluation

Right kidneys were formalin-fixed and paraffin-embedded. Samples were cut to a thickness of 5 µm, deparaffinized with xylene, and rehydrated in stepwise descending concentrations of ethanol and a single section per animal per method was analyzed. Sections were stained with Periodic Acid Schiff (PAS) and reviewed for the presence of comma-shaped and S-shaped immature bodies. Sections were also stained with *Lotus tetragonolobus* lectin and reviewed for histologic evidence of the nephrogenic zone via evaluation of presence of unstained tubule at the cortex. Mature glomerular number was estimated using radial glomerular counts. Briefly, a straight line was generated in a blinded fashion from the deepest cortex to the capsule in 3–5 nonoverlapping areas. The lines were placed at a magnification that glomeruli could not be distinguished to avoid bias. Mature glomeruli, defined as having capillary loops, were counted in an enlarged image. A secondary approach was also used to counter the challenges of getting the whole kidney in a mid-coronal plane (40). JRC (nephrologist) estimated the average number of layers of glomeruli across the entire section.

### Molecular evaluation of the nephrogenic niche

To evaluate molecular evidence of nephrogenesis, we performed immunofluorescence (IF) to assess for the presence of nephron progenitor cells (NPC) using SIX2, early epithelialization of the NPC using tight junction marker ZO-1, nascent nephron patterning using notch ligand Jagged 1 (JAG1) and canonical Wnt target/effector of Wnt signaling using transcription factor LEF1. IF was performed as previously described (6, 41) with the following exceptions: M.O.M. (Mouse on Mouse) Immunodetection Kit (Vector Laboratories) was used after initial blocking step for 1 h followed by Phosphate-buffered saline (PBS) washes × 2 for 2 min each, followed by antibody staining. Antibodies used include rabbit anti-SIX2 (11562-1-AP; Proteintech, 1:1,000), mouse anti-ZO-1 (339,100; Invitrogen, 1:400), rabbit anti-JAG1 (sc-8303; Santa Cruz, 1:100), rabbit anti-LEF1 (2230; Cell signaling, 1:100), and guinea pig Keratin 8 (Krt8) (GP-k8; Progen, 1:400). Images were acquired on the Nikon Ti2 Inverted SpectraX at 10× and 20× objectives. Tile scan images were obtained, and the cortical perimeter was measured using Elements 5.3.0 annotation tools. The SIX2^+^ (with and without ZO-1 co-staining), JAG1+, and LEF1+ niches were manually counted and divided per 1 mm of cortical distance of the entire cortical perimeter.

#### Adult kidney evaluation

At PN week 3, pups were weaned and all offspring were provided the 18% protein diet. Body weights were performed weekly beginning at weaning. At 6 wk of age, the adult LP and NP mice (n = 12 per diet per sex) underwent transdermal measurement of glomerular filtration rate (GFR) as previously reported (42). All mice were injected with horse spleen cationic ferritin (CF) for cationic ferritin enhanced-magnetic resonance imaging (CFE-MRI) and euthanized with tribromoethanol. Saline (0.9% NaCl) was infused via intracardiac injection to clear blood from the kidneys. The left renal artery was clamped, and perfusing fluid was switched to 10% formalin. The kidneys were collected and weighed. The right kidney was stored in formalin. The left kidney was stored in RNA later or flash frozen.

### Glomerular filtration rate

Each animal was sedated with isoflurane, and fur was removed from its back using an electric shaver followed by a depilation cream. A MediBeacon device was applied and secured with tape applied circumferentially around the mouse’s body to stabilize monitor placement without restricting breathing or movement. FITC-sinistrin (7.5 mg/100 g body weight; Fresenius-Kabi Austria, Linz) was injected into the tail vein of each mouse. Mice were observed for 90 min, during which they were conscious and able to move freely. MPD Studio software (Mannheim Pharma and Diagnostics) was used to create elimination kinetics curves. GFR was calculated as previously reported (42).

### Nephron number evaluation using ex vivo cationic ferritin enhanced MRI

Horse spleen cationic ferritin (CF, F7879; Sigma-Aldrich) was administered to each animal before euthanasia at a total dose of 5.75 mg per 100 g of body weight, delivered in two injections 90 min apart. Optimal MRI quality differed by sex, and thus male kidneys were collected 90 min after the last injection and female kidneys were collected 24 h after the last injection. The kidneys were stored in formalin but washed and imaged in phosphate-buffered saline (PBS). Kidneys were positioned in a customized container and imaged using a 9.4T MRI from Bruker. Images were acquired with a transmit-receive birdcage coil with inner diameter of 35 mm (Bruker Biospin) with the following parameters: 3D gradient recalled echo pulse sequence with a repetition time of 90 ms, echo time of 15 ms, field of view of 42 × 28 × 28 mm, matrix size of 768 × 512 × 512, flip angle 30˚ and isotropic spatial resolution of 55 × 55 × 55 µm. Images were viewed using Horos (version 3.3.6; www.horosproject.org) and segmented using 3D Slicer (https://www.slicer.org/). AutoGlom was used to measure glomerular number (N_glom_) and apparent median glomerular volume (aV_glom_) (43). Glomerular contrast (GC), a quantitative measure of the difference in intensity between glomerular regions and surrounding cortical areas, was used as a quality control metric (43). Only MRIs with GC >3 were included in the analysis (43).

### Histologic evaluation

Glomerular density, atubular glomeruli, proximal tubular fraction were measured as previously reported (42). Kidneys were prepared for histologic evaluation as previously described. To measure cortex area, glomerular density, and glomerular area, samples were stained with PAS. To measure atubular glomeruli (ATG) and proximal tubule (PT) fraction, samples were stained with *Lotus*. Slides were digitally scanned using the Grundium Ocus with a 20× objective (Grundium Ltd 2019). To measure the PT fraction, the Leica Microsystems CMS (Leica DM1000 LED) microscope was used to take 10–14 additional pictures at 40× in the subcapsular area. **Cortex area and glomerular density**: The histogram tool in Amira (Thermo Fisher Scientific software) was used to segment and measure the cortex area per 2D slide. Total absolute number of glomeruli per slide was manually counted. Glomerular density was quantified by the absolute number of glomeruli divided by cortex area. **Glomerular area**: All glomeruli in a 2D slide were individually segmented and measured using the histogram tool in 3D Slicer. **ATG and PT fraction**: To determine whether growth restriction resulted in proximal tubule pathology, both proximal tubular fraction and the integrity of the glomerulotubular junction were evaluated using *Lotus* staining. *Lotus* stains epithelial cells in the proximal tubule. Kidney sections underwent enzymatic digestion with proteinase K and subsequent biotinylated *Lotus* lectin stain (B1325; Vector Laboratories, 1:50 dilution), followed by a phosphate buffer wash and incubation with Vectastain ABC reagent3,3′diaminobenzidine. Sections were counterstained with methylene blue before dehydration. In ATG, where glomeruli are disconnected from proximal tubules, the epithelial cells of proximal tubule cells no longer take up *Lotus* staining. To quantify ATG, a *Lotus*-stained median sagittal kidney section was examined from each animal. Glomeruli were counted based on the presence or absence of *Lotus* staining within Bowman’s capsule. Atubular glomeruli were reported as the proportion of *Lotus*-positive glomeruli. As previously demonstrated (44), *Lotus*-positive glomerular fraction from a single section corresponds to normal glomerular fraction found in serial sections, and *Lotus*-negative glomeruli fraction from a single section corresponds to total glomeruli fraction with atrophic or absent tubules found in serial sections. To quantify PT fraction, the histogram tool in Amira was used to measure the proximal tubule area in 40× images, which was then divided by the total area to give PT fraction as previously reported (45).

### Podocyte density and glomerular area

Dual immunofluorescent immunostaining was performed with synaptopodin (Acris Antibodies Mouse Anti-Synaptopodin, Clone G1D4, BM5086, 1:10 dilution), WT-1 (Cat# MA5-32215; Thermo Fisher Scientific, 1:50 dilution), and appropriate secondary antibodies (Jackson Immunoresearch). Sodium citrate buffer was used for retrieval, and Hoeschst was applied to stain nuclei. Podocyte density and glomerular area were quantified using a protocol modified from Venkatareddy et al (46). Synaptopodin staining was used to quantify glomerular area, and Image J analytic software was used to quantify apparent mean WT+ podocyte nuclear diameter. Approximately 10 glomeruli per kidney were selected for quantification in an unbiased fashion representing glomeruli throughout the 2D section. Podocyte number per individual glomerular area was manually counted. Investigators performing the quantification were blinded to the animal group.

### Gene expression analysis

Total RNA was extracted from a quarter of the left kidney (n = 5/group) stored in RNAlater Stabilization solution using TRIzol (15596018; Invitrogen). The extracted RNA was further cleaned using the RNeasy MinElute Cleanup Kit (74204; Qiagen) with RNase-Free DNase Set (79254; Qiagen) to eliminate genomic DNA carryover. The extracted RNA was reconstituted with RNase-free water to a final volume of 50 μl and stored at –80 degrees until use. RNA concentration and quality were measured with a NanoDrop One/OneC Microvolume UV–Vis Spectrophotometer (Thermo Fisher Scientific), and the RNA Integrity Number (RIN) was obtained from the Genome Analysis and Technology Core at UVA using an Agilent 4200 TapeStation system. Non-directional bulk RNA-Seq was performed by Novogene Inc. Samples with low quantity (<10 ng) or low quality (RIN <4 and OD 260/280 <2.0) were eliminated from the analysis. The detailed sequencing statistics are shown in Table S10. Sequencing quality was assessed using FastQC (https://www.bioinformatics.babraham.ac.uk/projects/fastqc/). Low-quality reads were discarded prior to analysis. Obtained reads were adapter trimmed and then aligned to the GRCm39 mouse genome with the Gencode vM36 gene annotation using the STAR aligner (47). Transcripts were quantified using the *--quantMode GeneCount*s function of the STAR aligner, and normalization was applied, followed by differential expression assessed using the DESeq2 (48). Genes with at least ten reads per sample were included in the analysis. The RNA-sequencing data included in this publication have been deposited in NCBI’s Gene Expression Omnibus (49) and are accessible through GEO Series accession number GSE320330 (https://www.ncbi.nlm.nih.gov/geo/query/acc.cgi?acc=GSE320330).

Table S10. Detailed RNA sequencing statistics.

### Validation of molecular findings

Kidney sections from only male mice were prepared for immunohistochemical assessment with GFR⍺1 (NBP1-77043; Novus, 1:200) and a secondary antibody (Goat anti-rabbit secondary antibody, A-11034; Thermo Fisher Scientific, 1:250). GFR⍺1^+^ tubular area was quantified manually on ten sections using Amira, similar to PT fraction. Quantitative real-time polymerase chain reaction (tT-PCR) was performed on both male and female samples to validate bulk RNA-sequencing findings. Complementary DNA was synthesized according to the manufacturer’s instructions using the High-Capacity cDNA Reverse Transcription Kit (Thermo Fisher Scientific). qRT-PCR was performed using the CFX Opus 384 Real-Time PCR System with iTaq Universal SYBR Green Supermix (BIO-RAD) and each sample was run in duplicate. Primers were designed using the Primer3Plus (https://www.primer3plus.com/) and the expression levels for each candidate gene were normalized to *Gapdh*. Primer sequences are listed in Table S11.

Table S11. Primer sequences for gene validation with qRT-PCR.

### Statistical analysis

A sample size of 12 was calculated based on data from Hoppe et al (15) and our preliminary data. The effect size on glomerular number by MRI was ∼0.52 between the LP and NP male animals. Using an ⍺ = 0.05 and power of 80%, a sample size of n = 10/group was sufficient to detect the difference in N_glom_. However, this was increased by 20% to account for issues that may result in a low final number, such as mortality and issues with the kidney perfusions that are required for ex vivo CFE-MRI. Animals were stratified by sex *a priori* due to known sex differences. For bulk RNA-sequencing, a sample size of n = 5/group was used. Results are presented as median and standard deviations and were evaluated for statistical significance using Prism 9 (GraphPad software), with a *P* < 0.05 considered significant. Nonpaired *t* test or Mann-Whitney for nonnormally distributed data was used to compare LP to NP groups and male to female groups. Linear regression was used to determine the correlation of continuous variables and PN age.

Bulk RNA-sequencing was analyzed using both an unbiased (traditional bulk RNA-seq analysis) and a candidate-gene approach. Candidate genes were chosen *a priori* based on available literature. Individual genes were assigned to one of three categories: (1) kidney development, (2) nephron structure, or (3) pathways. Within categories, subcategories were defined as outlined in Table S12. Despite known roles across subcategories, each gene was only assigned to a single subcategory. Differentially expressed genes (DEGs) were identified using the Benjamini-Hochberg procedure for multiple comparison adjustments with a false discovery rate (FDR) set to *Q* = 0.05. Multiple comparison adjustments were made among genes within a subcategory.

Table S12. Candidate genes by category and subcategory.

## Supplementary Material

Reviewer comments

## Data Availability

The RNA-sequencing data included in this publication have been deposited in NCBI’s Gene Expression Omnibus (49) and are accessible through GEO Series accession number GSE320330 (https://www.ncbi.nlm.nih.gov/geo/query/acc.cgi?acc=GSE320330). Source data are included in the article or in supplementary materials (Tables S13 and S14). Table S13. Source data for adult cohort. Table S13. Source data for adult cohort. Table S14. Source data for neonatal cohort. Table S14. Source data for neonatal cohort.
