# Supplementary material for: Fetal growth restriction leads to prolonged nephrogenesis and gene dysregulation in the adult kidney
Source: Life Sci Alliance. 2026 Sep 23;9(11):e202603713. doi: 10.26508/lsa.202603713 (PMC13601832; doi:10.26508/lsa.202603713)
Supplement: Supplementary file 1 [file LSA-2026-03713_review_history.pdf]

Fetal growth restriction leads to prolonged nephrogenesis and gene dysregulation in the adult kidney

Sage Timberline, Rachel Dailey, Wyatt Johnson, Ayyappa Kameshwar, Jaya Isaac, Kimberly deRonde, Mark Conaway, Aleksandra Cwiek, Kevin Bennett, Edwin Baldelomar, Teng Li, Teresa Wu, Matthew Hoch, Meredith Schuh, Shalini Indugula, Yoshinori Seki, Masako Suzuki, Kimberly Reidy, and Jennifer Charlton
DOI: https://doi.org/10.26508/lsa.202603713

Corresponding author(s): Jennifer Charlton, University of Virginia

Review Timeline:

Submission Date:	2026-03-24
Editorial Decision:	2026-05-06
Revision Received:	2026-07-20
Editorial Decision:	2026-08-12
Revision Received:	2026-08-19
Accepted:	2026-08-25

Scientific Editor: Sarita Hebbar

Transaction Report:

(Note: With the exception of the correction of typographical or spelling errors that could be a source of ambiguity, letters and reports are not edited. The original formatting of letters and referee reports may not be reflected in this compilation.)

May 6, 2026

Re: Life Science Alliance manuscript #LSA-2026-03713-T

Dr. Jennifer R Charlton
University of Virginia
Box 800386
Charlottesville, VA 22903

Dear Dr. Charlton,

Thank you for submitting your manuscript entitled "Fetal growth restriction leads to prolonged nephrogenesis and persistent developmental gene dysregulation in the adult kidney" to Life Science Alliance. Your manuscript was reviewed by three expert reviewers whose comments are appended below.

As you will read, all the reviewers have highlighted significant concerns, majority of which centre around the incomplete descriptions of the methods and results, and a lack of validation of the RNAseq dataset. Based on the overall evaluation, we invite you to submit a revised manuscript addressing the reviewers' concerns.

We concur with all the reviewers that a revised manuscript must address all their concerns on scientific rigour and provide detailed description in the methods and results.
Reviewer 2 raises a valid point on the unexpected findings of Figure 2 in terms of the nephron endowment. We concur that a revised manuscript must resolve this concern with an experiment of your choice.
We agree with Reviewer 2 that you must provide a thorough validation of more of the differentially expressed genes from the RNA seq dataset either by immunochemistry or qPCR.

When submitting the revision, please include a letter addressing the reviewers' comments point by point. While a rebuttal must respond to all points in some form, additional experiments to resolve these points, other than indicated above, will not be required.

I would be happy to discuss the revision in more detail via email or phone/videoconferencing. Please let me know which option you prefer, if any.

To upload the revised version of your manuscript, please log in to your account: https://lsa.msubmit.net/cgi-bin/main.plex

You will be guided to complete the submission of your revised manuscript and to fill in all necessary information. Please get in touch in case you do not know or remember your login name.

The typical timeframe for revisions is three months. Please note that papers are generally considered through only one revision cycle, so strong support from the referees on the revised version is needed for acceptance.

While you are revising your manuscript, please also attend to the below editorial points to help expedite the publication of your manuscript. Please direct any editorial questions to the journal office.

Thank you for this interesting contribution to Life Science Alliance. We hope that the comments below will prove constructive as your work progresses, and we are looking forward to receiving your revised manuscript.

Sincerely,

Sarita Hebbar, PhD
Scientific Editor
Life Science Alliance
http://www.lsajournal.org

A. THESE ITEMS ARE REQUIRED FOR REVISIONS

-- A letter addressing the reviewers' comments point by point.

-- An editable version of the final text (.DOC or .DOCX) is needed for copyediting (no PDFs).

-- High-resolution figure, supplementary figure and video files uploaded as individual files: See our detailed guidelines for preparing your production-ready images, https://www.life-science-alliance.org/authors

-- Summary blurb (enter in submission system): A short text summarizing in a single sentence the study (max. 200 characters including spaces). This text is used in conjunction with the titles of papers, hence should be informative and complementary to the title and running title. It should describe the context and significance of the findings for a general readership; it should be written in the present tense and refer to the work in the third person. Author names should not be mentioned.

-- By submitting a revision, you attest that you are aware of our payment policies found here: https://www.life-science-alliance.org/copyright-license-fee

B. MANUSCRIPT ORGANIZATION AND FORMATTING:

Full guidelines are available on our Instructions for Authors page, https://www.life-science-alliance.org/authors

We encourage our authors to provide original source data, particularly uncropped/-processed electrophoretic blots and spreadsheets for the main figures of the manuscript. If you would like to add source data, we would welcome one PDF/Excel-file per figure for this information. These files will be linked as supplementary "Source Data" files.

IMPORTANT: It is Life Science Alliance policy that if requested, original data images must be made available. Failure to provide original images upon request will result in unavoidable delays in publication. Please ensure that you have access to all original microscopy and blot data images before submitting your revision.

Reviewer #1 (Comments to the Authors (Required)):

This report describes a study in which kidneys were studied in offspring of dams that had been subjected to a low protein diet that was rendered isocaloric to control diet by balancing with sucrose. Dams were fed 8% protein diet versus 18% protein diet ad lib through gestation and to weaning. Cohorts of pups were studied during the first postnatal week and another cohort was weaned, fed regular 18% protein chow and analyzed at 6 weeks of age. RNAseq was performed on the adult group. The histological data is convincingly interpreted and shows some interesting differences between feeding groups, such as an extension of expression od developmental genes in kidneys after birth in the 8% protein group. Differential gene analysis suggests perturbations in the GDNF and retinoic acid pathways which are both essential for kidney development.

In my opinion, the data is valuable but it is difficult to interpret because there is no explanation of the feeding model. For example, while feed was isocaloric it is not described if the animals ate similar amounts. If intake indeed was equivalent, could effects on gestation be due to high sucrose rather than low protein? The results section needs to include a proper rationale for the feeding strategy and references to supporting papers. Since this is not a specialty journal, it is important that the nutritional aspects of the study are properly explained to readers.

Reviewer #2 (Comments to the Authors (Required)):

This manuscript aims to further characterize a well-used model of low nephron endowment and provides some of the first published transcriptional differences between adult mice with nutritionally altered nephron endowments. The paper is largely descriptive with little hypothesis driven investigation, but that likely fits within the scope of this journal. The authors could significantly build on this work using the RNASeq data to design interesting experiments.

Specific Comments below:
Figure 1:

There are atypical features discussed in the results like correlation of growth trends and differences in the intercepts, which is difficult to interpret. It would be more prudent to objectively show the weights, highlight which timepoints are different, and leave additional interpretation to the readers.

Figure 2:

Please indicate on the images what you are considering the nephrogenic zone, a Six2 niche, a JAG1 Niche, and a LEF1 niche.

Include results of your statistical analysis on the figures.

These findings are not what would be expected given the final outcome in nephron endowment. Do the authors have any hypotheses about this? Perhaps the prolonged RV/SSB structures are not actually completing differentiation. One could consider TUNEL staining to assess whether those cells may be undergoing apoptosis. Or do daily nephron counts around the time of six2 jag1 niche depletion to see if prolonged presence of those structures is leading to additional differentiated structures at those later timepoints.

Figure 3:

The same data appear to be presented in both a table and graphs. I would recommend choosing the best way to represent your data and moving forward with that.

Figure 3 is an aggressive characterization of a frequently used model of low nephron endowment, but I'm left wondering about the significance of many of these findings. What does glom density mean clinically, or proximal tubule fraction, or ATG? If you feel that it's important to include these findings, please elaborate on the meaning in the discussion.

RNASeq data. Why did you only validate GFra1 with immunohistochemistry, but didn't validate any other differentially expressed genes, even with something simple like qPCR?

For most readers of this manuscript, the RNASeq results will be the most interesting data. I may include more of those results in the paper instead of the supplement.

Reviewer #3 (Comments to the Authors (Required)):

Timberline and colleagues provide a report detailing a study of maternal protein-restriction on mice and the impact on kidney development, structure and function. The authors acknowledge that many studies have been published in this area. Timberline and colleagues provide validation of previously published findings (reduced nephron number, reduced kidney function, altered gene expression). This study does provide more detail on the postnatal window in mice during which nephrogenesis occurs, and how it differs in pups exposed to maternal protein-restriction. However, the manuscript does not advance the field or add any significantly novel findings in a well-published model.
The decision to reject the manuscript is based on the following observations:
- Unclear number of litters used. The authors should be commended for the care taken when producing their animals by exposing the grand-maternal line to the control NP diet. Additionally, more detail required to discuss potential limitations of reduced genetic diversity (in a model with limited genetic diversity). It appears that all experimental animals would have been close genetic relatives?
- Unclear what postnatal diet was used for experimental animals. It has been well-documented the deleterious impact of 'catch-up' growth.
- Report n values for sex and for litters. It is commendable that the authors have investigated both sexes. This should be reported for each experiment where it occurred, and justified when it was not (simply stating there was not significant difference may not suffice when you can report the values).
- The authors state, "To control for the duration of gestation, an additional litter of pups from a timed pregnancy for each diet group was used." It is unclear what this means and what scientific evidence it provided to the study.
- Unclear which kidney, or whether it was the same kidney in all animals, was used to determine the nephrogenic window. In addition, it is unclear how many sections were analysed per kidney.
- A discussion is required to explore the possibility that the experimental design (ie litter sizes were significantly reduced from postnatal day 2 - 7) may introduce a variable similar to that in cross-fostering models.
- Details are missing in the Methods section regarding how GFR was calculated (Page 6, Line 208). This appears unfinished.
- The findings regarding changes in gene expression in adult kidneys requires more justification. The authors note that these genes are involved in kidney development, but the analysis has occurred in adult kidneys. What do these data mean for the 'adult molecular phenotype'?
- All tables and images should include n values.
- Results section should provide more descriptive data where appropriate.
- The aim stated for this paper was: 'This work aims to determine the duration of nephrogenesis and define the adult molecular phenotype associated with growth restriction, laying the groundwork for understanding the increased risk of CKD in those with low nephron endowment'. Therefore, more work and focus should be dedicated to the molecular data.

Dear Reviewers and Editors,
We appreciate the positive and constructive feedback in your review of our manuscript, "Fetal growth restriction leads to prolonged nephrogenesis and persistent developmental gene dysregulation in the adult kidney". We have addressed each concern point by point below in *italics* and have coded the edits within the manuscript by editor or reviewer number (E, or R1, R2, and R2). We have provided additional experiments to address the unexpected findings in Figure 2 and to validate the RNA Sequencing data. We believe our revisions, guided by the expertise of the Reviewers, have significantly improved the manuscript. We appreciate the opportunity to share this revised manuscript.

Sincerely,
Jennifer Charlton, MD, MSc

Editor:

1. Reviewer 2 raises a valid point on the **unexpected findings of Figure 2** in terms of the nephron endowment. We concur that a revised manuscript must resolve this concern with an experiment of your choice.

Please see below (R2.4) for response.

2. We agree with Reviewer 2 that you must provide a **thorough validation** of more of the differentially expressed genes from the RNA seq dataset either by immunochemistry or qPCR.

Please see below (R2.7) for response.

When submitting the revision, please include a letter addressing the reviewers' comments point by point. While a rebuttal must respond to all points in some form, additional experiments to resolve these points, other than indicated above, will not be required.
Below we have addressed each concern point by point below in italics and have coded the edits within the manuscript.

Reviewer #1:

This report describes a study in which kidneys were studied in offspring of dams that had been subjected to a low protein diet that was rendered isocaloric to control diet by balancing with sucrose. Dams were fed 8% protein diet versus 18% protein diet ad lib through gestation and to weaning. Cohorts of pups were studied during the first postnatal week and another cohort was weaned, fed regular 18% protein chow and analyzed at 6 weeks of age. RNAseq was performed on the adult group. The histological data is convincingly interpreted and shows some interesting differences between feeding groups, such as an extension of expression od developmental genes in kidneys after birth in the 8% protein group. Differential gene analysis suggests perturbations in the GDNF and retinoic acid pathways which are both essential for kidney development.

1. In my opinion, the data is valuable but it is difficult to interpret because there is **no explanation of the feeding model**. For example, while feed was isocaloric it is not described if the animals ate similar amounts. If intake indeed was equivalent, could effects on gestation be due to high sucrose rather than low protein? The results section needs to include a **proper rationale** for the feeding strategy and references to supporting papers. Since this is not a specialty journal, it is important that the nutritional aspects of the study are properly explained to readers.

We appreciate the reviewer's questions about the model. We did not track intake or maternal body weight because entering the cages daily or handling the pregnant mice is stressful and could significantly impact the results of the study. We have now included a more detailed explanation and several references to this well-established model in the Methods sections. We agree that there is the potential that the additional sucrose could affect our findings. High sucrose diets have been reported

to have more effect on the vasculature, and while we cannot entirely decouple the effects of the low protein and higher sucrose, our phenotype closely mirrors historic low protein literature where complex starches were used to maintain calories. We have included these limitations in the Discussion.

Reviewer #2:

This manuscript aims to further characterize a well-used model of low nephron endowment and provides some of the first published transcriptional differences between adult mice with nutritionally altered nephron endowments. The paper is largely descriptive with little hypothesis driven investigation, but that likely fits within the scope of this journal. The authors could significantly build on this work using the RNASeq data to design interesting experiments.

Specific Comments below:

1. Figure 1: There are atypical features discussed in the results like correlation of growth trends and differences in the intercepts, which is difficult to interpret. It would be more prudent to objectively show the weights, highlight which timepoints are different, and leave additional interpretation to the readers.

We have removed the emphasis on growth trend correlations and intercept differences from both the figure and the Results section, indicating the time points at which statistically significant differences were observed and allowing the readers to interpret the growth patterns directly from the data. In addition, we have revised Figure 1 to include a new graph illustrating body weight trends across the full postnatal period to provide a clearer visualization of overall growth trajectories.

2. Figure 2: Please indicate on the images what you are considering the nephrogenic zone, a Six2 niche, a JAG1 Niche, and a LEF1 niche.

We have edited the Figure 2 as suggested.

3. Include results of your statistical analysis on the figures.

We have now included the results of the statistical analysis in the figures.

4. These findings are not what would be expected given the final outcome in nephron endowment. Do the authors have any hypotheses about this? Perhaps the prolonged RV/SSB structures are not actually completing differentiation. One could consider TUNEL staining to assess whether those cells may be undergoing apoptosis. Or do daily nephron counts around the time of six2 jag1 niche depletion to see if prolonged presence of those structures is leading to additional differentiated structures at those later timepoints.

We appreciate the reviewer's insightful comment. We have now estimated the number of mature glomeruli using radial glomerular counts, counting the number of mature glomeruli along a line from the inner cortex to the capsule. We found the LP group started with fewer mature glomeruli at PN3, and by PN6 they had more glomeruli than at PN3. Along with the molecular data, this suggests that the LP pups have a slower but longer duration of nephrogenesis. We have included these new data along with methods and references in the revised manuscript.

5. Figure 3: The same data appear to be presented in both a table and graphs. I would recommend choosing the best way to represent your data and moving forward with that.

To eliminate redundancy, we have retained the graphical presentation of the data in the main manuscript and moved the corresponding table to the Supplement (sTable 7). The legend for Figure 3 has also been expanded to provide relevant detail for the data presented in the graphs. We believe

these revisions improve the clarity of the main figure while preserving access to the complete numerical data for readers who wish to examine it in greater detail.

6. Figure 3 is an aggressive characterization of a frequently used model of low nephron endowment, but I'm left wondering about the significance of many of these findings. What does glom density mean clinically, or proximal tubule fraction, or ATG? If you feel that it's important to include these findings, please elaborate on the meaning in the discussion.

We agree that the functional and clinical significance of the histologic findings was underdeveloped in the original submission. We have added a paragraph to the Discussion that interprets the findings in terms of functional consequence and, where applicable, its relationship to our transcriptomic data.

7. RNASeq data. Why did you only validate GFra1 with immunohistochemistry, but didn't validate any other differentially expressed genes, even with something simple like qPCR?

We have conducted a validation study using 14 samples, including 9 genes in the female group and 6 genes in the male group. Our qRT values strongly correlate with our bulk RNA-Sequencing normalized counts (p<0.0001). These data are now included in Supplemental Figure 2. We also include qRT data on a few of the relevant genes in sFigure 2.

8. For most readers of this manuscript, the RNASeq results will be the most interesting data. I may include more of those results in the paper instead of the supplement.

We appreciate the reviewer's thoughtful suggestion and agree that the RNA sequencing findings are a central component of the manuscript. In response, we have moved the volcano plots from the Supplementary Materials to the main figures to provide readers with a more comprehensive overview of the differential gene expression results. The accompanying Results section has also been revised to incorporate more discussion of these data in the main text.

Reviewer #3 (Comments to the Authors (Required)):
Timberline and colleagues provide a report detailing a study of maternal protein-restriction on mice and the impact on kidney development, structure and function. The authors acknowledge that many studies have been published in this area. Timberline and colleagues provide validation of previously published findings (reduced nephron number, reduced kidney function, altered gene expression). This study does provide more detail on the postnatal window in mice during which nephrogenesis occurs, and how it differs in pups exposed to maternal protein-restriction. However, the manuscript does not advance the field or add any significantly novel findings in a well-published model. The decision to reject the manuscript is based on the following observations:

1. Unclear number of litters used. The authors should be commended for the care taken when producing their animals by exposing the grand-maternal line to the control NP diet.

We have included litter number in sTable 3.

2. Additionally, more detail required to discuss potential limitations of reduced genetic diversity (in a model with limited genetic diversity). It appears that all experimental animals would have been close genetic relatives?

Unrelated females and males were mated for the litters included in the study. We included 4 litters used in the nephrogenesis portion of the study and 3-4 litters used for the adult portion. This is now included in sTable 3. We used an outbred strain, CD-1 mice, that are generally considered to have moderate genetic heterogeneity with more diversity than C57Bl/6.

3. Unclear what postnatal diet was used for experimental animals. It has been well-documented the deleterious impact of 'catch-up' growth.

We thank the reviewer for highlighting this important point. We have revised the Methods section to clarify that all offspring were transitioned to the standard control diet following weaning, regardless of prenatal dietary exposure. We have also expanded the Results section to note that low protein offspring did not exhibit catch-up growth during the study period. We agree that postnatal growth trajectories are an important consideration when interpreting developmental programming outcomes, and these revisions provide additional context for our findings.

4. Report n values for sex and for litters. It is commendable that the authors have investigated both sexes. This should be reported for each experiment where it occurred, and justified when it was not (simply stating there was not significant difference may not suffice when you can report the values).

We thank the reviewer for the positive feedback and helpful suggestion. We have now detailed the animal counts for each assessment (including number of animals by sex) and the numbers of litters contributing to each experiment in sTable 2 and 3, respectively. We have also included a statement both in the Methods section and in the Figure legends directing readers to this information.

5. The authors state, "To control for the duration of gestation, an additional litter of pups from a timed pregnancy for each diet group was used." It is unclear what this means and what scientific evidence it provided to the study.

We thank the reviewer for the opportunity to clarify this methodological detail. Using litters from timed pregnancies was necessary to precisely characterize the timing of nephrogenesis, as differences in gestation length between NP vs LP pregnancies could confound interpretation of nephrogenesis timing data. In other words, pups of the same postnatal age could represent different stages of renal development if gestation length differed between groups. This clarification has been added to the methods section.

6. Unclear which kidney, or whether it was the same kidney in all animals, was used to determine the nephrogenic window. In addition, it is unclear how many sections were analysed per kidney.

We have edited the Methods section to address these issues.

7. A discussion is required to explore the possibility that the experimental design (ie litter sizes were significantly reduced from postnatal day 2 - 7) may introduce a variable similar to that in cross-fostering models.

We agree that litter size is a recognized determinant of nutrient availability per pup, as utilized in cross-fostering models, and that reduction in litter size over postnatal days 2-7 was inherent in our design. Importantly, starting litter sizes were comparable between groups (LP: 8-13; NP: 9-13), and pups were collected on the same schedule from both LP and NP litters (1-2 pups per litter per day), so the progressive reduction in litter size was equivalent across LP and NP groups at every time point. This clarification has been added to the Methods section, and is also further discussed in the Discussion and limitations.

8. Details are missing in the Methods section regarding how GFR was calculated (Page 6, Line 208). This appears unfinished.

We have revised the Methods section to include the appropriate reference describing the methodology, and removed the incomplete sentence.

9. The findings regarding changes in gene expression in adult kidneys requires more justification. The authors note that these genes are involved in kidney development, but the analysis has occurred in adult kidneys. What do these data mean for the 'adult molecular phenotype'?

Developmental pathways contribute to the response of the kidney to injury and regulate kidney epithelial differentiation, extracellular matrix remodeling, repair, fibrosis, and maladaptive injury responses. Reviews by Little and by Edeling et al. discuss activation of developmental programs in adult kidney repair/fibrosis, and low-protein diet studies support developmental programming effects on nephron endowment and later kidney risk. We have included these articles in our Discussion.

10. All tables and images should include n values.

We have revised tables and images to include n values, with an additional reference to sTable 2 for detailed counts.

11. Results section should provide more descriptive data where appropriate.

We agree that the Results section would benefit from more descriptive data and have revised it, specifically adding the body and kidney weights for pups and adults in addition, GFR, Nglom, PT and ATG's are included for the adults.

12. The aim stated for this paper was: 'This work aims to determine the duration of nephrogenesis and define the adult molecular phenotype associated with growth restriction, laying the groundwork for understanding the increased risk of CKD in those with low nephron endowment'. Therefore, more work and focus should be dedicated to the molecular data.

We agree with the reviewer. In the revision, we have moved the volcano plots into the main portion of the manuscript and we have added new experiments to validate our molecular findings.

August 12, 2026

RE: Life Science Alliance Manuscript #LSA-2026-03713-TR

Dr. Jennifer R Charlton
University of Virginia
Box 800386
Charlottesville, VA 22903

Dear Dr. Charlton,

Thank you for submitting your revised manuscript entitled "Fetal growth restriction leads to prolonged nephrogenesis and gene dysregulation in the adult kidney".

Your revised manuscript was evaluated by one of the original reviewers whose comments are appended below. As you will read, this reviewer has acknowledged that your revised manuscript has largely addressed their previous concerns.

In line with their evaluation, we would be happy to publish your paper in Life Science Alliance pending resolution of final revisions necessary to meet our formatting guidelines.

MANUSCRIPT ORGANIZATION AND FORMATTING:

To avoid unnecessary delays in the acceptance and publication of your paper, please read the following information carefully. Full guidelines are available on our Instructions for Authors page, https://www.life-science-alliance.org/authors

Submission of a paper that does not conform to Life Science Alliance guidelines will delay the acceptance of your manuscript.

-Please indicate the scale bar size in the legend for Figure 2. Kindly include scale bar information for the boxed region if shown at a higher magnification.
-Please integrate all supplementary methods description into the 'Materials and Methods' section of the main ms file.
-Please upload all figure files as individual ones, including the supplementary figure files; all figure legends should only appear in the main manuscript file. After that, please remove the file containing supplementary information.
-Thank you for providing a statement confirming that all experiments were performed in accordance with relevant guidelines and regulations. Kindly provide an affiliation for the Animal Care and Use Committee approving procedures involving animals (lines 145-46).
-Please be sure that the authorship listing and order are correct and match between the system and the manuscript file.
-Please consult our manuscript preparation guidelines https://www.life-science-alliance.org/manuscript-prep and make sure your manuscript sections are in the correct order.
-Please remove information about word and character count from the title page. Please also remove ETOC and TRANSLATIONAL STATEMENT be removed from the manuscript file.
-Please be sure that the sections of the manuscript file are labeled in accordance with the LSA requirements, e.g., Disclosure Statement should be labeled as "Conflict of Interest", etc.
-Please add your main, supplementary figure, and table legends to the main manuscript text after the references section.
-For consistency, please use the following callout format throughout the manuscript: Table S1, Table S2, ..., Table S8, and Figure S1, Figure S2, etc.
-Please add callouts for Figures S1A-C and S2A-C to your main manuscript text.
-Please add the X and Bluesky handles of your host institute/organization, as well as your own, and/or one of the authors, in our system.We encourage our authors to provide original source data, particularly uncropped/-processed electrophoretic blots and spreadsheets for the main figures of the manuscript. If you would like to add source data, we would welcome one PDF/Excel-file per figure for this information. These files will be linked as supplementary "Source Data" files.
-Please be sure that the authorship listing and order is correct.

It is Life Science Alliance policy that if requested, original data images must be made available to the editors. Failure to provide original images upon request will result in unavoidable delays in publication. Please ensure that you have access to all original data images prior to final submission.

If you are planning a press release on your work, please inform us immediately to allow informing our production team and scheduling a release date.

We welcome submissions of potential cover images for the issue of LSA in which your work would appear. If you have high

quality images associated with this work, please feel free to email these, with a caption, to the journal office.

LSA encourages authors to provide a 30-60 second video where the study is briefly explained. These videos will be appear embedded with the manuscript online at Life Science Alliance, and on social media to promote the published paper and authors (for examples, see https://docs.google.com/document/d/1-UWCfbE4pGcDdcgzcmiuJl2XMBJnxKYeqRvLLrLSo8s/edit?usp=sharing). Corresponding or first-authors are welcome to submit the video. Please submit only one video per manuscript. The video can be emailed to contact@life-science-alliance.org

FINAL FILES:

To upload the final version of your manuscript, please log in to your account: https://lsa.msubmit.net/cgi-bin/main.plex
You will be guided to complete the submission of your revised manuscript and to fill in all necessary information. Please get in touch in case you do not know or remember your login name.

The following items are required for acceptance.

-- An editable version of the final text (.DOC or .DOCX) is needed for copyediting (no PDFs).

-- High-resolution figure, supplementary figure and video files uploaded as individual files: See our detailed guidelines for preparing your production-ready images, https://www.life-science-alliance.org/authors

-- Summary blurb (enter in submission system): A short text summarizing in a single sentence the study (max. 200 characters including spaces). This text is used in conjunction with the titles of papers, hence should be informative and complementary to the title. It should describe the context and significance of the findings for a general readership; it should be written in the present tense and refer to the work in the third person. Author names should not be mentioned.

The license to publish form must be signed before your manuscript can be sent to production. A link to the license to publish form will be available to the corresponding author only. Please take a moment to check your funder requirements.

Reviews, decision letters, and point-by-point responses associated with peer-review at Life Science Alliance will be published, alongside the manuscript. If you do want to opt out of having the reviewer reports and your point-by-point responses displayed, please let us know immediately.

Thank you for your attention to these final processing requirements. Please revise and format the manuscript and upload materials as soon as you are able.

Thank you for this interesting contribution to the literature. We look forward to publishing your paper in Life Science Alliance.

Sincerely,

Sarita Hebbar, PhD
Scientific Editor
Life Science Alliance
http://www.lsajournal.org

Reviewer #2 (Comments to the Authors (Required)):

The authors have satisfactorily addressed my concerns.

Dear LSA Editors,
We appreciate the positive and constructive feedback in your review of our manuscript, "Fetal growth restriction leads to prolonged nephrogenesis and gene dysregulation in the adult kidney". We have addressed each concern point by point below in *italics*. We appreciate the opportunity to share this revised manuscript.

Sincerely,
Jennifer Charlton, MD, MSc

1. Please indicate the scale bar size in the legend for Figure 2. Kindly include scale bar information for the boxed region if shown at a higher magnification.
    a. *Completed*
2. Please integrate all supplementary methods description into the 'Materials and Methods' section of the main ms file.
    a. *Completed*
3. Please upload all figure files as individual ones, including the supplementary figure files; all figure legends should only appear in the main manuscript file. After that, please remove the file containing supplementary information.
    a. *Completed*
4. Thank you for providing a statement confirming that all experiments were performed in accordance with relevant guidelines and regulations. Kindly provide an affiliation for the Animal Care and Use Committee approving procedures involving animals (lines 145-46).
    a. *We have added that this approval was obtained at the University of Virginia.*
5. Please be sure that the authorship listing and order are correct and match between the system and the manuscript file.
    a. *Completed*
6. Title should be <100.
    a. *We have adjusted the title to comply.*
7. Inclusion of a summary blurb
    a. *Completed*
8. Changed order of methods and materials to after discussion
    a. *We changed the order and renumbered all of the supplemental tables to be in the order they now appear in the manuscript.*
9. Please remove information about word and character count from the title page.
    a. *completed*
10. Please also remove ETOC and TRANSLATIONAL STATEMENT be removed from the manuscript file.
    a. *Completed*
11. Please be sure that the sections of the manuscript file are labeled in accordance with the LSA requirements, e.g., Disclosure Statement should be labeled as "Conflict of Interest", etc.
    a. *Completed*
12. Please add your main, supplementary figure, and table legends to the main manuscript text after the references section.
    a. *Completed*
13. For consistency, please use the following callout format throughout the manuscript: Table S1, Table S2, ..., Table S8, and Figure S1, Figure S2, etc. Please add callouts for Figures S1A-C and S2A-C to your main manuscript text.
    a. *Adjusted.*
14. Please add the X and Bluesky handles of your host institute/organization, as well as your own, and/or one of the authors, in our system.
    a. *Completed*
15. We encourage our authors to provide original source data, particularly uncropped/-processed electrophoretic blots and spreadsheets for the main figures of the manuscript. If you would like to add source data, we would welcome one PDF/Excel-file per figure for this information. These files will be linked as supplementary "Source Data" files.

   a. *We have provided the source data for the adult and neonatal experiments in Tables S13 and S14, respectively, and have referenced these tables in the Data Availability section. During preparation of these source-data files, we identified an inconsistency in the reported animal number. We corrected the animal number and updated Figure 3g for consistency with the underlying source data. These corrections did not affect the statistical results, interpretation, or conclusions of the study.*

16. Please be sure that the authorship listing and order is correct.
    a. *Completed*

August 25, 2026

RE: Life Science Alliance Manuscript #LSA-2026-03713-TRR

Dr. Jennifer R Charlton
University of Virginia
Box 800386
Charlottesville, VA 22903

Dear Dr. Charlton,

Thank you for submitting your Research Article entitled "Fetal growth restriction leads to prolonged nephrogenesis and gene dysregulation in the adult kidney". We apologise for the delay in communicating a decision due to editor availability issues.

It is a pleasure to let you know that your manuscript is now accepted for publication in Life Science Alliance. Congratulations on this interesting work.

The final published version of your manuscript will be deposited by us to PubMed Central upon publication.

Your manuscript will now progress through copyediting and proofing. It is journal policy that authors provide original data upon request.

Your article will publish open access upon publication under a CC-BY license.

Reviews, decision letters, and point-by-point responses associated with peer-review at Life Science Alliance will be published, alongside the manuscript. If you do want to opt out of having the reviewer reports and your point-by-point responses displayed, please let us know immediately.

IMPORTANT: If you will be unreachable at any time, please provide us with the email address of an alternate author. Failure to respond to routine queries may lead to unavoidable delays in publication.

Scheduling details will be available from our production department. You will receive proofs shortly before the publication date. Only essential corrections can be made at the proof stage so if there are any minor final changes you wish to make to the manuscript, please let the journal office know now.

DISTRIBUTION OF MATERIALS:
Authors are required to distribute freely any materials used in experiments published in Life Science Alliance. Authors are encouraged to deposit materials used in their studies to the appropriate repositories for distribution to researchers.

You can contact the journal office with any questions, contact@life-science-alliance.org

Again, congratulations on a very nice paper. I hope you found the review process to be constructive and are pleased with how the manuscript was handled editorially. We look forward to future exciting submissions from your lab.

Sincerely,

Sarita Hebbar, PhD
Scientific Editor
Life Science Alliance
http://www.lsajournal.org
